# Rapid Assessment of Virtually Synthesizable Chemical Structures via Support Vector Machine Models

**DOI:** 10.1002/minf.70000

**Published:** 2025-07-21

**Authors:** Yuto Iwasaki, Tomoyuki Miyao

**Affiliations:** ^1^ Graduate School of Science and Technology Nara Institute of Science and Technology Nara Japan; ^2^ Data Science Center Nara Institute of Science and Technology Nara Japan

**Keywords:** chemical structure generation, combinatorial synthesis, quantitative structure‐activity relationships, support vector machine, virtual synthesis

## Abstract

Support vector machine (SVM) and support vector regression (SVR) are widely used for building quantitative structure–activity relationship models for small‐ and medium‐sized datasets. Although SVM and SVR models can efficiently predict compound activity, evaluating billions of molecules remains challenging, which sometimes occurs when screening the virtual molecules derived through virtual synthesis. Herein, we present an SVM‐/SVR‐based method for screening virtually synthesizable molecules based on their reactants. The proposed method employs a combination of reactant‐wise kernel functions for fast evaluation without sacrificing prediction accuracy. Tested on 120 small molecular activity datasets against 10 macromolecule targets, the proposed SVR models with data augmentation worked equally to standard SVR models with the Tanimoto kernel. As a demonstration, exhaustive 6.4 × 10^12^ reactant combinations were evaluated by an SVR model within 8 days on a single desktop computer, enabling large‐scale screening without sampling.

## Graphical abstract

A method is proposed to rapidly assess many virtual compounds consisting of reactants using a support vector machine (regression) model with a tailored kernel function.

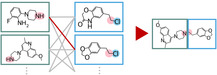

## 1 Introduction

In data‐driven chemistry, quantitative structure–activity relationship (QSAR) models are widely used to computationally screen compound databases for identifying bioactive compounds [1, 2], which will be the subject of subsequent wet laboratory experiments. This screening—virtual screening (VS)—can even suggest novel molecules as long as the screened database includes novel chemical structures. Several methods for identifying novel bioactive compounds have adopted this approach [3, 4], where a screening database was a focused library of bioactive‐like virtual molecules generated by a molecular generative model [5]. Generally, a molecular generative model can generate novel molecules that are structurally similar to the compounds used for model training. Some methods can incorporate constraints or conditions to generate molecules with desirable properties [6, 7, 8, 9–10]. Subsequently, QSAR models can be applied to these virtual molecules as additional filters.

An important premise of the VS of a novel molecule database is that virtual molecules in the database must be synthesized for subsequent experimental testing. A systematic evaluation of virtual molecules generated by generative models revealed that the synthesizability of these molecules is similar to that of the compound dataset used for model training when a distribution‐based generative model was used [11]. For example, a variational autoencoder model trained on a dataset extracted from the ChEMBL database [12] generated 60% of synthesizable virtual molecules on the basis of ASKCOS, an open‐source software framework containing data‐driven retrosynthesis models [13]. Although this success rate seems promising, the availability of starting materials, the accessibility of reaction conditions, cost, and scalability strongly influence whether the proposed chemical structures can be synthesized.

To overcome the issue of synthesizability, methods for synthesis‐oriented molecular structure generation have been proposed [14, 15, 16, 17–18]. Most of the proposed methods incorporate template‐based forward virtual synthesis to produce virtual molecules through virtual synthesis paths. Swanson et al. proposed a Monte Carlo tree search‐based molecular generation approach to generate synthesizable antibiotic candidates [14]. In their study, virtual molecules were created through 13 virtual reactions from a set of 132 479 building blocks, the predicted antibacterial activity based on QSAR models served as scores for the search. The chemical space (searching space) in their method was estimated at approximately 30 billion molecules, whose number was intractable with standard computers, and an optimization approach was introduced instead of evaluating exhaustive virtual molecules.

Support vector machine (SVM) and support‐vector regression (SVR) are widely used for QSAR modeling [19, 20–21]. They can construct nonlinear QSAR models via the kernel trick: projecting descriptors into the Hilbert space through a kernel function, followed by linear modeling. Commonly used kernel functions include the Tanimoto and radial basis function (RBF) functions to define the similarity between data points [22]. Directly representing the similarity principle: similar molecules exhibiting similar properties, makes this approach still appealing, particularly for modeling using small‐ to medium‐sized datasets. Thus, developing a method to propose novel synthesizable molecules based on an SVM/SVR‐based activity prediction model is practically important.

Herein, we propose a method to generate novel and synthesizable virtual molecules with desirable activity (property) on the basis of an SVM/SVR model and retrosynthesis analysis. Proposed virtual molecules are constructed through a single‐step reaction from available compounds and are thus virtually synthesizable. The SVM/SVR model is built with a combination of reactant‐wise kernel functions, enabling the fast screening of sets for paired reactants. The number of exhaustive combinations of reactant pairs easily reaches a large figure, for example, 10^10^ (100 000 compounds for each reactant). The proposed method aims to quickly assess a vast number of reactant combinations without sampling: exhaustive evaluation of the virtual reactant combinations based on an SVM/SVR model. In laboratory‐scale experiments, feasible reactions are often predetermined or restricted by available equipment and facilities. The proposed method would also be beneficial in that situation because it focuses on a specific reaction.

However, it was unclear whether the prediction accuracy of the SVM model built on reactants was comparable to that of the model built on products, that is, the compounds themselves. To investigate this, we systematically compiled 60 datasets against 10 macromolecular targets, each consisting of a single reaction type, and evaluated the prediction accuracy for these datasets. A data augmentation strategy was introduced to address incorrectly guided reactant splitting. As a workflow to propose virtual molecules, the proposed method was compared with a combinatorial virtual molecule generation approach based on Thompson sampling [23].

## 2 Computational Methods

### 2.1 Exhaustive Virtual Molecule Evaluation

**FIGURE 1:**
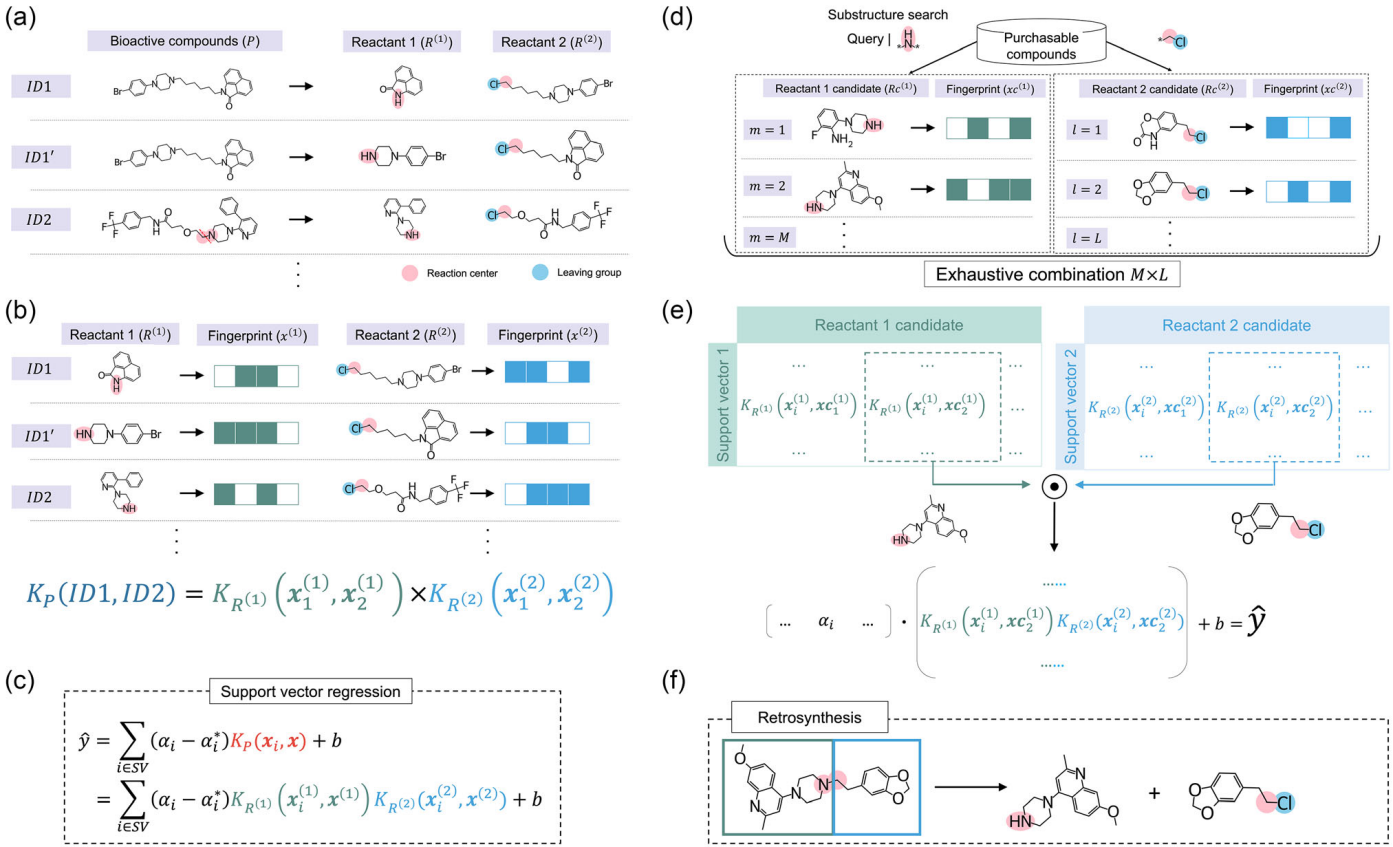
Overview of the study. (a) On the basis of a specific reaction rule (e.g., an SN2 reaction forming a tertiary amine), compounds (products) are retrosynthesized into two precursors (reactants), and the reactants form a dataset. A virtual reactant pair (ID1′) was introduced as data augmentation. (b) Fingerprints of the reactants were calculated, and kernel function values between two compounds (reactions) were determined as the product of reactant component‐wise kernel functions. If the reaction template dissects a product in different ways, the set of virtual reactants is included in the training dataset. (c) A support vector regression model is constructed using the product kernel function. (d) Reactant candidates are obtained via substructure search from a purchasable compound database to propose virtual molecules on the basis of the synthesis path. (e) For each reactant component (e.g., amine), a kernel matrix of the support vectors and candidate reactants is prepared, and the predicted y values are derived by a linear operation using the values. (f) The retrosynthesis of the proposed virtual compounds confirmed the validity of the compounds.

Figure 1 shows the proposed workflow for structure generation using retrosynthesis and an SVR‐based activity prediction model. The activity prediction model is built on a dataset of compounds synthesized through a single reaction type. The reaction can be either the actual reaction for producing the compounds or a virtual synthesis based on a retrosynthesis analysis of the compounds. In this study, a retrosynthesis model was used to form a set of compounds generated from the same reaction (*vide infra*). Since the same reaction is applied to the compounds, an organized table can be made where rows are compounds and columns are reactants (Figure 1a). The SVR model uses a product kernel (PK) function of reactant component fingerprints to predict the compound potency, enabling an independent calculation of kernel values for each reactant component (Figure 1b,c). If the reaction template can dissect a product in different ways from the actual reactants, a set of virtually possible reactants is also added to the training dataset as data augmentation. This operation expands the domain of applicability using valid reactant pairs. To generate virtual molecules, reactant candidates are screened from a purchasable compound database on the basis of the reaction center queries (Figure 1d). To efficiently predict activity values for virtual molecules synthesized from the screened reactants, the kernel value matrices of candidate reactants are formed (Figure 1e), where rows are support vectors (SVs) and columns are candidate reactants. Because the formula for predicting a y value of the SVR model is a linear combination of the products between regression coefficients and kernel values against the SVs, inner product and intercept summation operations produce a predicted y value. Virtual compounds (reactants) are filtered out on the basis of their predicted y values. The synthesizability of the virtual molecules is confirmed by the retrosynthesis prediction model (Figure 1f). The last step can be replaced with any model to evaluate the feasibility of the virtual reaction, particularly when using an actual synthesis‐based compound dataset in the initial step (Figure 1a).

### 2.2 Molecular Representation

The extended connectivity fingerprint with a diameter of 4 (ECFP4) [24] was used as a molecular representation. The feature set generated by ECFP4 was folded into an 8192‐bit vector with the *GetMorganFingerprintAsBitVect* function in the RDKit library [25] (version 2023.9.5), an open‐source cheminformatics library. The reaction centers of reactants are marked with isotopes so that they take different hash numbers from other atoms.

### 2.3 Activity (Potency) Prediction Models

#### 2.3.1 SVR with the PK Function

An SVR model was developed to rapidly calculate prediction *y* values for many reactant combinations using the PK of Tanimoto kernels for reactants. SVR uses the loss function of an L1 norm with an epsilon‐insensitive tube and a penalty term representing the complexity of the model. SVR, in combination with the Tanimoto kernel function, has been extensively studied in potency prediction and is generally regarded as a standard approach because of its high prediction accuracy [26, 27]. The PK between two compounds, ID_1_ and ID_2_, corresponding to two pairs of reactants (***x***_1_^(1)^, ***x***_1_^(2)^) and (***x***_2_^(1)^, ***x***_2_^(2)^), is written as

(1) $$K\left(ID_{1},ID_{2}\right)=K_{T}\left(x_{1}^{\left(1\right)},x_{2}^{\left(1\right)}\right)\times K_{T}\left(x_{1}^{\left(2\right)},x_{2}^{\left(2\right)}\right)$$

where *x*_i_^(j)^ represents the fingerprint of the *j*‐th reactant for the *i*‐th compound (*i* ∈ {1, …, *N*}, *j* ∈ {1, 2}), *N* is the number of compounds (reaction pairs) in the dataset, and *K*_T_ is the Tanimoto kernel function. This modeling method was termed SVR‐PK for simplicity. The hyperparameters C and epsilon in the SVR module were optimized on the basis of the 5‐fold cross‐validation prediction accuracy in GridSearchCV provided by Scikit‐learn [28] (version 1.4.1.post1).

#### 2.3.2 Models for Comparison

Potency prediction from a pair of reactants can be achieved using any kernel function. In this study, SVR was combined with the summation of the two Tanimoto kernel (SK) functions, termed SVR‐SK, and simply concatenating the reactant ECFP4s followed by the Tanimoto kernel application, termed SVR‐concatECFP, were used as comparison methods. Compound ECFP4 was also used as the baseline, which is termed the SVR baseline. Furthermore, a recently proposed graph neural network‐based deep learning model, MolCLR [29], was used. A pretrained version of the MolCLR model was fine‐tuned for each reaction dataset, and the best model based on the validation loss was used for activity prediction for the test dataset. Each training dataset was randomly split into 90% for training and 10% for validation for fine‐tuning.

### 2.4 Screening Reactants from a Purchasable Compound Database

Reactant candidates were screened from a purchasable compound database to generate virtual molecules through the same reaction employed to produce training compounds. The reaction queries were represented as reaction SMARTS focusing on the reaction centers. Only molecules with a single reaction center were selected as candidates to avoid regioselectivity issues.

The screened reactant candidates were further evaluated on the basis of the outputs of the SVR‐PK model. The number of possible reactant combinations is the product of the numbers for the first and second reaction components. If 100 000 candidates are screened for each component, then 10^10^ virtual molecules should be evaluated in the activity prediction‐based screening. Thus, a computationally efficient way of calculating prediction y values was necessary. SVR‐PK was employed to ease the combinatorial explosion. The predicted y value for the compound ***x*** = (***x*** ^(1)^, ***x*** ^(2)^) consisting of reactants ***x*** ^(1)^ and ***x*** ^(2)^ can be written as:

(2) $$\hat{y}\left(x\right)=\sum_{i\in SV}\left(\alpha_{i}-\alpha_{i}^{*}\right)K_{T}\left(x^{\left(1\right)},x_{i}^{\left(1\right)}\right)\times K_{T}\left(x^{\left(2\right)},x_{i}^{\left(2\right)}\right)+b$$

where *α*s are dual coefficients, SV represents the support vectors of the model, and b is the intercept term. By preparing the tables consisting of kernel function values between the reactants and SVs, (Equation 2) requires 2 × *n*SV + (*n*SV − 1) + 1 basic arithmetic operations (Figure 1e), where nSV is the number of support vectors. On the basis of the exhaustive evaluation of the candidate reactants, a focused library containing only virtual molecules with desired predicted potencies can be created and further scrutinized with synthetic availability as explained in the **Synthesizability of Virtual Molecules** section. In this study, the focused library size was set to 10 000 due to the high computational demand of the comparison method for sampling.

#### 2.4.1 Screening Method for Comparison

Klarich et al. proposed a scalable sampling approach from sets of reactants for multiple components [23]. Their method is to independently apply Thompson sampling to the reaction component and repeatedly samples a pair of reaction candidates for both components with the best scores from the belief distributions. The model scores the selected pairs, and the belief distributions are updated in a Bayesian manner. For this Thompson sampling approach, the warmup phase requires evaluating n counterpart reactants to make belief distributions. In this study, the number of sampling molecules was 10 000, which aligns with the exhaustive structure generation with our approach.

### 2.5 Synthesizability of Virtual Molecules

A focused library containing virtual molecules with purchasable reactants is further scrutinized in terms of reaction feasibility. After removing molecules containing elements other than C, N, O, F, P, S, Cl, Br, and I, having a molecular weight of greater than 800, and having rings consisting of more than 7 heavy atoms, retrosyntheses of the virtual molecules were conducted to check whether the proposed reactants matched the purchasable reactants (screened reactants) (see the **Retrosynthesis Prediction Model** section). The virtual molecules passing this filter became the proposal molecules by our method.

### 2.6 Retrosynthesis Prediction Model

When the reaction of producing training compounds is not specified, the retrosyntheses of compounds are required to create a set of reactants for the same reaction type. We used a retrosynthesis method proposed by Coley et al. [30]. The method mimics chemists’ approach to identifying precursors on the basis of similarity between a query and target reactions. First, similarity values between a target compound and products from a reaction database of USPTO‐50k are calculated, and the top 100 similar reactions are considered. The compound is then cleaved into reactants (retrosynthesis) by applying the inverse reaction template extracted from the reaction of each of the products. The similarity values between the reactants obtained and the reactants of the template reaction are subsequently calculated. Finally, the product of the similarity values for the reactants and the product determines whether the retrosynthesis is eligible or not. In this study, the top 10 reactions were obtained for each target compound. If the 10th and 11th reactions had the same score, both reactions were selected. For some compounds, fewer than 10 reactions were obtained because of the failure of the precise reaction template matching.

### 2.7 Method Implementation

The SVR models were built with *sklearn.svm, SVR* module of the scikit‐learn library [28] (version 1.4.1.post1). Custom kernels, PK, and SK were defined in‐house scripts. Substructure matching for identifying candidate reactants was implemented with RDKit [25]. Reactant sampling via Thompson sampling was conducted with the Python program provided at https://github.com/PatWalters/TS. Retrosynthesis by the method of Coley et al. was conducted with the Python program package provided at https://github.com/connorcoley/retrosim.

### 2.8 Bioactive Compound Datasets

The proposed method was validated through the process of proposing virtual molecules for various biological targets. To compile training datasets, compounds active against human macromolecules with measured pK_i_ (the logarithm of reciprocal inhibition constant) values were downloaded from the ChEMBL database [12] (version 31). Only high‐confidence assays with direct target molecules were selected, and bioactive datasets against ten macromolecules were determined on the basis of K‐medoids clustering results, which were further organized by reaction types as a result of retrosynthesis analysis.

For each of the ten datasets, bioactive compounds were retrosynthesized into two reactants. Decomposition into more than two components was ignored in this study. The reaction centers and leaving groups were labeled with isotopic numbers of 1000 and 900, respectively, to distinguish the reaction centers from other atoms. The reactants were then grouped by the reaction templates to form reaction datasets. The reaction datasets were then curated by selecting reactions whose reactants consisted of the elements (C, N, O, F, S, P, Cl, Br, and I), having a molecular weight less than or equal to 800, and containing sizes of the smallest set of shortest rings (SSSR) less than or equal to 7. Furthermore, outlier reactions whose reactant(s) were outside of 1.5 times the interquartile range from either quantile in the heavy atom counts were eliminated, and small datasets of fewer than 100 compounds (reactions) were excluded. After the curation, the ten most populated reaction datasets were selected for each target, and then smaller reaction datasets that overlapped 60% with other reaction datasets were removed. The reaction datasets with the number of reactant pairs and compounds (products) for each target are provided in Table 1, and the corresponding reaction templates (reaction SMARTS) and the patent IDs from which the reaction templates were extracted are provided in Table S1 of Supporting Information. The reaction datasets can be identified by unique dataset IDs from 1 to 60 in Table 1.

**TABLE 1:** Reaction dataset profiles.

Reaction dataset ID	Reactant pairs^a^	Compounds^b^	CHEMBL ID	Target description
1	215	209	214	5‐hydroxytryptamine receptor 1A
2	406	254		
3	441	283		
4	515	407		
5	2504	2141		
6	379	318	217	D (2) dopamine receptor
7	3954	3624		
8	157	153	218	Cannabinoid receptor 1
9	168	145		
10	225	206		
11	252	233		
12	254	248		
13	438	432		
14	1266	1201		
15	1416	707		
16	114	114	222	Sodium‐dependent noradrenaline transporter
17	140	136		
18	152	146		
19	165	142		
20	189	128		
21	198	177		
22	438	288		
23	162	136	224	5‐hydroxytryptamine receptor 2A
24	233	147		
25	393	230		
26	1948	1801		
27	139	122	225	5‐hydroxytryptamine receptor 2C
28	167	117		
29	222	127		
30	308	255		
31	453	438		
32	144	144	228	Sodium‐dependent serotonin transporter
33	184	173		
34	189	156		
35	228	158		
36	432	365		
37	561	394		
38	648	610		
39	102	101	238	Sodium‐dependent dopamine transporter
40	147	144		
41	155	147		
42	197	175		
43	316	219		
44	120	119	240	Potassium voltage‐gated channel subfamily H member 2
45	131	130		
46	148	126		
47	163	122		
48	172	128		
49	202	175		
50	257	248		
51	361	345		
52	771	369		
53	355	297	261	Carbonic anhydrase 1
54	368	286		
55	395	341		
56	399	383		
57	442	411		
58	814	549		
59	1630	785		
60	2193	1640		

a The number of reactant pairs for the dataset.

b The number of compounds (products) in the dataset.

### 2.9 Reactant Screening Database

A purchasable compound database for screening reactants was prepared, which was extracted from the eMolecules screening database [31]. A total of 18 177 461 screening compounds from the eMolecules database were filtered by removing duplicates on the basis of canonical SMILES strings, resulting in 17 935 690 compounds. After applying the same curation process for preparing the reaction datasets, 17 718 146 compounds remained, forming the purchasable compound database.

### 2.10 Evaluation

The proposed virtual molecule generation workflow was evaluated from two aspects: the prediction accuracy of the activity prediction model with SVR‐PK and the exhaustive screening of candidate reactants followed by virtual molecule generation.

### 2.11 Evaluation of Activity Prediction Models

Predictions from reactants instead of the chemical structure of a compound (product) might reduce the prediction accuracy due to the loss of information about the whole chemical structure, particularly using the PK or SK, which independently considers the similarity within each reaction component. Thus, the prediction accuracy of SVR‐PK was compared with that of SVR‐SK, SVR‐concatECFP, the SVR baseline, and MolCLR models for the reaction datasets against the ten macromolecules. Two types of training–test data splitting strategies were used to evaluate the prediction accuracy. The evaluation metrics used were the coefficient of determination (*R* ^2^), the root mean squared error (RMSE), and the mean absolute error (MAE), defined as follows:

(3) $$R^{2}=1-\frac{\sum_{i=1}^{n}{\left(y_{i}-{\hat{y}}_{i}\right)}^{2}}{\sum_{i=1}^{n}{\left(y_{i}-\bar{y}\right)}^{2}}$$

(4) $$\text{RMSE}=\sqrt{\frac{1}{n}\sum_{i=1}^{n}{\left(y_{i}-{\hat{y}}_{i}\right)}^{2}}$$

(5) $$\mathrm{MAE}=\frac{1}{n}\sum_{i=1}^{n}\left|y_{i}-{\hat{y}}_{i}\right|$$

where *y*_i_ and ${\hat{y}}_{i}$ are the observed and predicted values for the *i*‐th compound, $\bar{y}$ is the mean of the *y* values for the samples, and *n* is the number of samples.

Predicted potency values for the different reactant pairs that form the same compound (product) were averaged to produce a single prediction potency value for the compound. In this way, reactant and compound‐based models were evaluated per the same compounds in the test datasets.

#### 2.11.1 Training and Test Dataset Splitting

To fairly evaluate the models’ generalizability, two splitting strategies, product‐ and reactant‐based splitting, were applied to prepare training and test datasets.

In product‐based splitting, compounds (products) in each reaction dataset were randomly divided into training and test datasets at 6:4. Test compounds with a similarity value of more than or equal to 0.8 were removed. Then, the reactants were also divided into the corresponding classes of products (training or test datasets) to ensure that a tuple of a reactant pair and the corresponding product belonged to the same class. Although this splitting ensures that there are no overlapping products between the training and test datasets, some reactants might belong to both the training and test datasets.

In reactant‐based splitting, no overlapping reactants exist between the training and test datasets, let alone no overlapping products. Since different compounds might be derived from the same reaction component, product‐based splitting is insufficient to fully evaluate the generalizability of prediction from reaction components, which is a similar situation using a combinatorial dataset [32, 33]. To make training and test datasets from each reaction dataset, reaction components were aligned on grid points where each axis represented a reaction component; a grid point of (r_1_, r_2_) was a reaction of r_1_ as the first reaction component and r_2_ as the second. The order of the reactants for each reaction component was determined as follows. First, for each reactant component *i* (1 or 2), reactants were sorted in descending order in the frequency of appearance of the reactants: 1 to *n* (r_i1_, r_i2_, … r_i (*n*−1)_, r_in_). For the reactant component 1, reactants were reordered using an alternating pattern: starting from the most frequent, then the least frequent, followed by the second most frequent, the second least frequent, and so on (r_11_, r_1n_, r_12_, r_1(n−1)_, …). This ordering helps evenly distribute commonly used reactants across the grid space to avoid regional bias. For the reactant component 2, reactants were simply ordered in descending frequency. Then, the set of grid points was divided into four regions by vertical and horizontal lines, and the positions of these lines were adjusted so that the ratio of the number of grid points in the left lower and right upper regions was as close to 6:4 as possible. The reactions residing in the left lower region became a training dataset, and those in the right upper region, a test dataset.

#### 2.11.2 Data Augmentation

**FIGURE 2:**
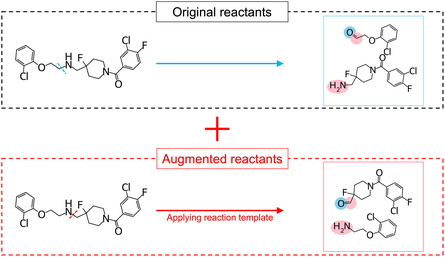
Training data augmentation by virtually dissecting a product (compound).

To expand the domain of applicability of reactant‐based models, the reaction template of dissecting a product to form a set of reactants is systematically applied to the product to obtain various reactant sets. These virtual reactants are also used for model training as a data augmentation method (Figure 2).

### 2.12 Evaluation of Virtual Molecule Generation

Three reaction datasets for different reactions and target macromolecules were selected to evaluate the generation workflow. The exhaustive molecule generation method was compared with the generation approach using Thompson sampling [23]. Since the Thompson sampling approach could not rank all possible combinations, 10 000 virtual molecules (reactions) were focused on, followed by applying retrosynthesis to finalize the proposed molecules. Since prediction from the chemical structure of a compound is supposed to be more accurate than prediction from reactants, the SVR baseline model was used to predict potencies for the Thompson sampling approach. For our proposed method, which relies on reactants, the exhaustive combinations of candidate reactants were evaluated using the SVR‐PK model. The top 10 000 virtual molecules were selected from the exhaustive combinations, followed by retrosynthesis. The screening process was evaluated in terms of the run time and number of screened compounds. The generated molecules were evaluated in terms of the number of unique scaffolds (diversity) and the range of predicted activity on the basis of the SVR baseline model. Scaffolds were identified with the *MurckoScaffold* module of RDKit [25].

## 3 Results and Discussion

### 3.1 Potency Prediction Accuracy

**FIGURE 3:**
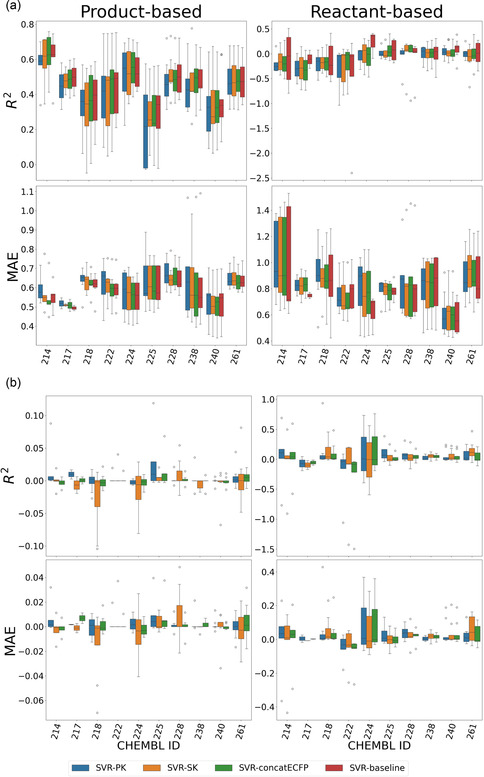
Prediction accuracy of the SVR models and the effect of data augmentation. (a) For each target, the prediction accuracy (*R* ^2^ or MAE) is reported in a box plot. Accuracies for product‐based splitting are shown on the left, and those for reactant‐based splitting are shown on the right. (b) The difference in prediction accuracy (*R* ^2^ or MAE) between the data augmentation and non‐augmentation strategies is shown in a boxplot (data augmentation – non‐augmentation in *R* ^2^, and non‐augmentation – data augmentation in the MAE). The data‐augmented strategy used generated virtual reactants by applying the reaction template to training products at different reaction centers from which the original training dataset was created.

The prediction accuracy of the SVR models is summarized in terms of *R* ^2^ and MAE in Figure 3a for the 60 reaction datasets. According to the overall scale of the metric values, prediction for reactant‐based split datasets was more challenging than that for product‐based split datasets. Compared with the four SVR modeling approaches, SVR‐PK, SVR‐SK, SVR‐concatECFP, and the SVR baseline, the SVR baseline slightly outperformed the other approaches. Overall, MolCLR showed lower prediction accuracy (comparison with SVR‐based models is provided in Figure S1, Tables S6 and S8 of Supporting Information), possibly due to small dataset sizes. In product‐based splitting, the SVR baseline showed the highest prediction ability on average for 5 out of the 10 targets, followed by SVR‐concatECFP (3 targets), SVR‐SK (1 targets), SVR‐PK (1 target), and MolCLR (0 target). In reactant‐based splitting, SVR‐PK showed the highest prediction ability on average for 4 out of 10 targets, followed by SVR‐baseline (3 targets), MolCLR (2 target), SVR‐SK (1 target), and SVR‐concatECFP (0 target). As shown in Table 2, in the target‐wise prediction accuracy comparison, only one out of the 10 targets favored the SVR baseline over the SVR‐PK, with a *statistically significant difference* (*p value* below 0.05) in product‐based splitting. Furthermore, in reactant‐based splitting, the SVR baseline did not yield significantly higher prediction accuracy than the other reactant‐based approaches. Therefore, the SVR modeling approaches using reactant‐based kernel functions with data augmentation did not impair the prediction accuracy of the original SVR models when all the compound information was used. Surprisingly, for datasets produced via reactant‐based splitting, which involved more rigorous validation due to the lack of overlap between the training and test datasets in terms of both reactant and product, the proposed SVR‐PK outperformed the SVR baseline, possibly because of the data‐augmentation effect, which is briefly explained in the next section.

**TABLE 2:** Statistical testing of the target wise R^2^ difference from the SVR baseline. For each data‐splitting strategy, p‐values calculated by the two‐sided Wilcoxon signed‐rank test in R^2^ are reported between (1) SVR‐PK and SVR‐baseline, (2) SVR‐SK and SVR‐baseline, and (3) SVR‐concatECFP and SVR‐baseline.

CHEMBL ID	Product based			Reactant based		
	(1)	(2)	(3)	(1)	(2)	(3)
214	0.63	0.63	0.63	0.44	0.31	0.63
217	0.50	0.50	0.50	0.50	1.00	0.50
218	0.08	0.02	0.31	0.31	0.20	0.20
222	0.22	0.11	0.81	1.00	0.63	0.63
224	1.00	0.63	0.63	0.50	0.25	0.25
225	0.63	0.81	0.63	0.25	0.88	0.38
228	0.02	0.69	0.16	0.63	0.63	0.19
238	0.31	0.63	1.00	0.88	0.63	0.63
240	0.20	0.91	1.00	0.65	0.65	0.91
261	0.55	0.64	0.55	0.16	0.81	0.81

The effect of introducing possible virtual reactant sets into the training dataset was evaluated (data augmentation) in terms of the difference in prediction accuracy (Figure 3b). On the basis of the average performance difference, prediction accuracy was improved for 8 targets (28 reaction datasets) for SVR‐PK, for 3 targets (19 reaction datasets) for SVR‐SK, and for 3 targets for SVR‐concatECFP (18 reaction datasets) when product‐based splitting was employed. Similarly, the prediction accuracy improved for 7 targets (30 reaction datasets) for SVR‐PK, for 6 targets (33 reaction datasets) for SVR‐SK, and for 5 targets for SVR‐concatECFP (26 reaction datasets) when reactant‐based splitting was used. Model prediction accuracies for all datasets for both the training and test datasets are provided in Tables S2–S9 of Supporting Information.

### 3.2 Example Case of the Effect of Data Augmentation

**FIGURE 4:**
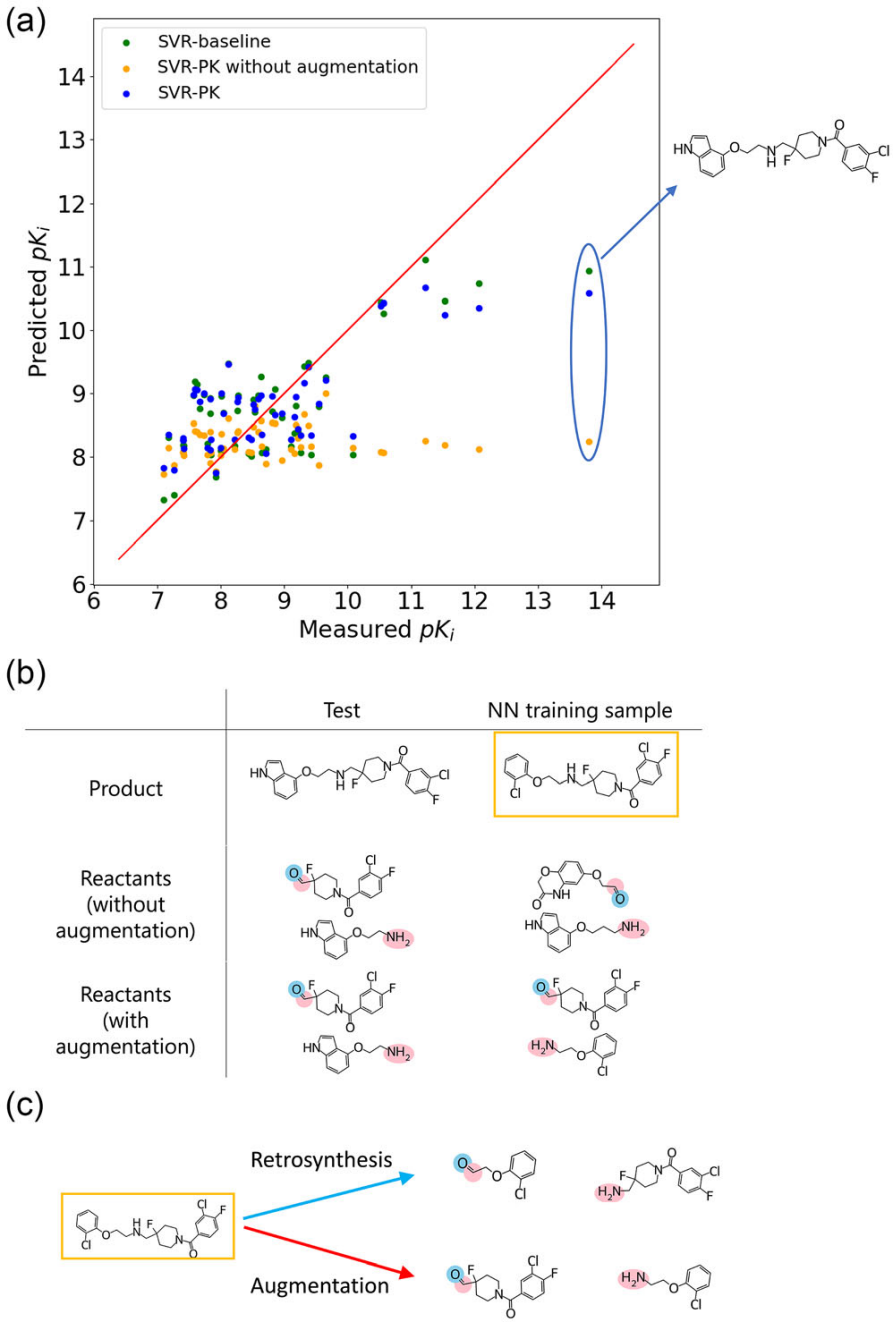
Data augmentation improved the prediction accuracy through nearest neighbor (NN) compound adjustments for the Reaction dataset ID: 2. (a) *y*‐*y* plots for the test dataset using the three approaches: the SVR baseline (green), the SVR‐PK without augmentation (orange) and the SVR‐PK (blue), where the blue oval corresponds to a test compound specified in the structural formula. (b) The NN compound (pairs) in the training dataset for the test compound (blue square), where red circles are reaction centers and blue circles are leaving groups. (c) The training reactants for the NN training compound (yellow) in (b) were generated by retrosynthesis through the blue decomposition path, whereas in data augmentation, the decomposition specified by the red line was conducted, producing a reactant set similar to that of the test reactants.

The effect of introducing virtual reactants by dissecting products using the same reaction template but different reaction centers is explained in Figure 4. Figure 4a shows the predicted versus observed value plot for the Reaction dataset ID: 2 using three approaches: the SVR baseline (product‐based), SVR‐PK without data augmentation (reactant‐based without data augmentation), and SVR‐PK with data augmentation (reactant‐based with data augmentation). Apparently, the reactant‐based model without data augmentation showed poor prediction accuracy, particularly for the region with higher potency. Data augmentation eased the gap in prediction accuracy between product‐ and reactant‐based approaches. The test compound showing the largest prediction error using the reactant‐based approach without data augmentation (circled in blue) is shown in Figure 4b. In Figure 4b, the nearest neighbor (NN) compound and pair of reactants are also shown as a matrix. The NN compound was an analog of the test compound with a Tanimoto similarity value of 0.75. The reactants for the NN compound did not generate a similar reactant pair for the test compound. The reaction template for this dataset was ([CX4 + 0H2R0:1]‐[NX3 + 0H1R0:2])>>[NX3 + 0H2R0:2]. [O]=[CX3 + 0H1R0:1], and two dissecting points existed for the product compound containing a secondary amine bonded to two SP3 carbon atoms with two hydrogens (Figure 4c). It is generally difficult to know which one of the pairs of reactants was prepared in advance. Without data augmentation, test reactants sometimes could not find a similar training reactant set, even when the test compound is an analog of training compounds.

### 3.3 Virtual Molecule Generation

**FIGURE 5:**
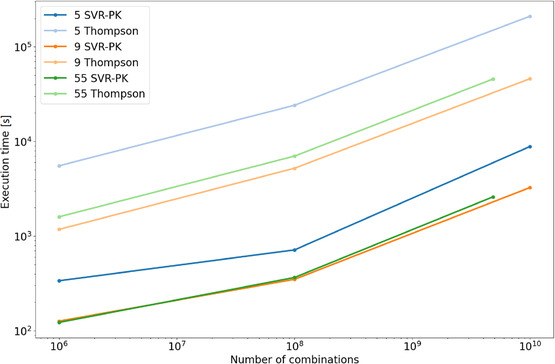
Execution time for screening. Machine: CPU; Intel (R) Xeon (R) Platinum 8273CL CPU @ 2.20 GHz, RAM; 576 GB.

For reaction datasets ID 5, 9, and 55, virtual molecule generation exercises were carried out. The reaction template and target macromolecule for ID 5 was ([CX4 + 0H2R0:1]‐[NX3 + 0H0R:2])>>[Cl]‐[CX4 + 0H2R0:1]. [NX3 + 0H1R:2] and 5‐hydroxytryptamine receptor 1A, for ID 9 ([cX3 + 0H0R:1]‐[OX2 + 0H0R0:2])>>[Cl]‐[cX3 + 0H0R:1]. [OX2 + 0H1R0:2] and cannabinoid receptor 1, and for ID 55 ([CX3 + 0H0R0:1]‐[cX3 + 0H0R:2])>>[Cl]‐[CX3 + 0H0R0:1].[cX3 + 0H1R:2] and carbonic anhydrase 1. The *R* ^2^ value for the reaction dataset ID 5 by SVR‐PK was 0.59, that by the SVR baseline was 0.61, those for ID 9 were 0.34 and 0.35, and those for ID 55 were 0.32 and 0.34. By screening the eMolecules database using the reaction templates, pairs of reactants were prepared. The numbers of candidate reactants for these datasets were (238 927, 548 350) for ID 5, (2 628 949, 2 444 769) for ID 9, and (48 342, 533 522) for ID 55. Accordingly, the numbers of possible combinations were 1.3 × 10^11^, 6.4 × 10^12^, and 2.6 × 10^10^ for IDs 5, 9, and 55, respectively. Each set of reactant combinations was screened via the SVR‐PK‐based ranking method and Thompson sampling. To compare the screening times of the methods, three sets of reactant pairs were prepared, (10^3^, 10^3^), (10^4^, 10^4^), and (10^5^, 10^5^), and the execution times for screening the three datasets were monitored, as reported in Figure 5. Apparently, the SVR‐PK‐based method screened pairs faster than the Thompson sampling approach. The evaluation of the exhaustive combinations was also carried out. When evaluating 6.4 × 10^12^ combinations (ID 9) by the SVR‐PK model, it took 8 days using a single computer with a CPU: Intel (R) Xeon E5−2640 v4 @ 2.40 GHz and 128 GB of RAM. However, it took too much time to handle those combinations by the Thompson sampling‐based approach. To compare the generated compounds under the same experimental conditions, the number of combinations was capped at 10^10^ (10^5^ for each reactant component) in this study.

For the proposed method, the top 1 million pairs were selected on the basis of the ranking by the SVR‐PK model, followed by the selection of the top 10 000 virtual molecules by reranking via the SVR baseline model, assuming that the SVR baseline model yielded higher prediction accuracy, although this assumption was not always supported by our benchmark evaluation results (**Potency Prediction Accuracy** section). Table 3 summarizes the numbers of compounds generated by the proposed virtual molecule generation workflow compared with the Thompson sampling‐based approach. Overall, SVR‐PK and Thompson sampling selected different compound sets, representing a few overlapping compounds between the two methods. SVR‐PK selected a higher lowest potency of the selected compounds, while no clear difference was observed in the highest potency. In terms of the number of eligible products passing the retrosynthesis filter, SVR‐PK proposed more highly predicted potent compounds, possibly because the selected compound a few scaffolds, as shown in Table 3. The number of unique scaffolds generated by these methods implied that Thompson sampling produced more diverse virtual molecules than the SVR‐PK method did. However, when dissecting the molecules into reactants, the Thompson sampling method used a few reactants for Reactant 1, three reactants for ID 5, and one reactant for IDs 9 and 55. The diversity in terms of the number of scaffolds in the Thompson sampling came from Reactant 2 in this study.

**TABLE 3:** Screening compounds (products). For each reaction dataset, (10^5^, 10^5^) reactant pairs were investigated using a single computer with an Intel (R) Xeon (R) Platinum 8273CL CPU, and 576 GB of RAM.

Reaction dataset ID	Method	Explored combinations	Screened products	**Eligible products** a	** Range of predicted pK**_**i**_ b	Intersections	Unique scaffolds	Reactant 1		Reactant 2	
								Unique reactants	Unique scaffolds	Unique reactants	Unique scaffolds
5	**SVR‐PK**	1.0 × 10^10^	10 000	9871	8.49–9.57	217	3556	1550	229	187	101
	**Thompson sampling**	–	10 000	9084	7.13–9.57		6538	3	3	5411	3782
9	**SVR‐PK**	1.0 × 10^10^	10 000	9125	7.12–7.60	0	1784	82	75	3686	128
	**Thompson sampling**	–	10 000	7358	6.87–7.49		2968	1	1	7358	1949
55	**SVR‐PK**	4.8 × 10^9^	10 000	5420	6.83–7.32	186	987	73	27	4205	553
	**Thompson sampling**	–	10 000	2790	6.34–7.59		1203	1	1	2790	1088

a Products that passed the curation and retrosynthesis filter.

b Predicted values by the SVR baseline model.

**FIGURE 6:**
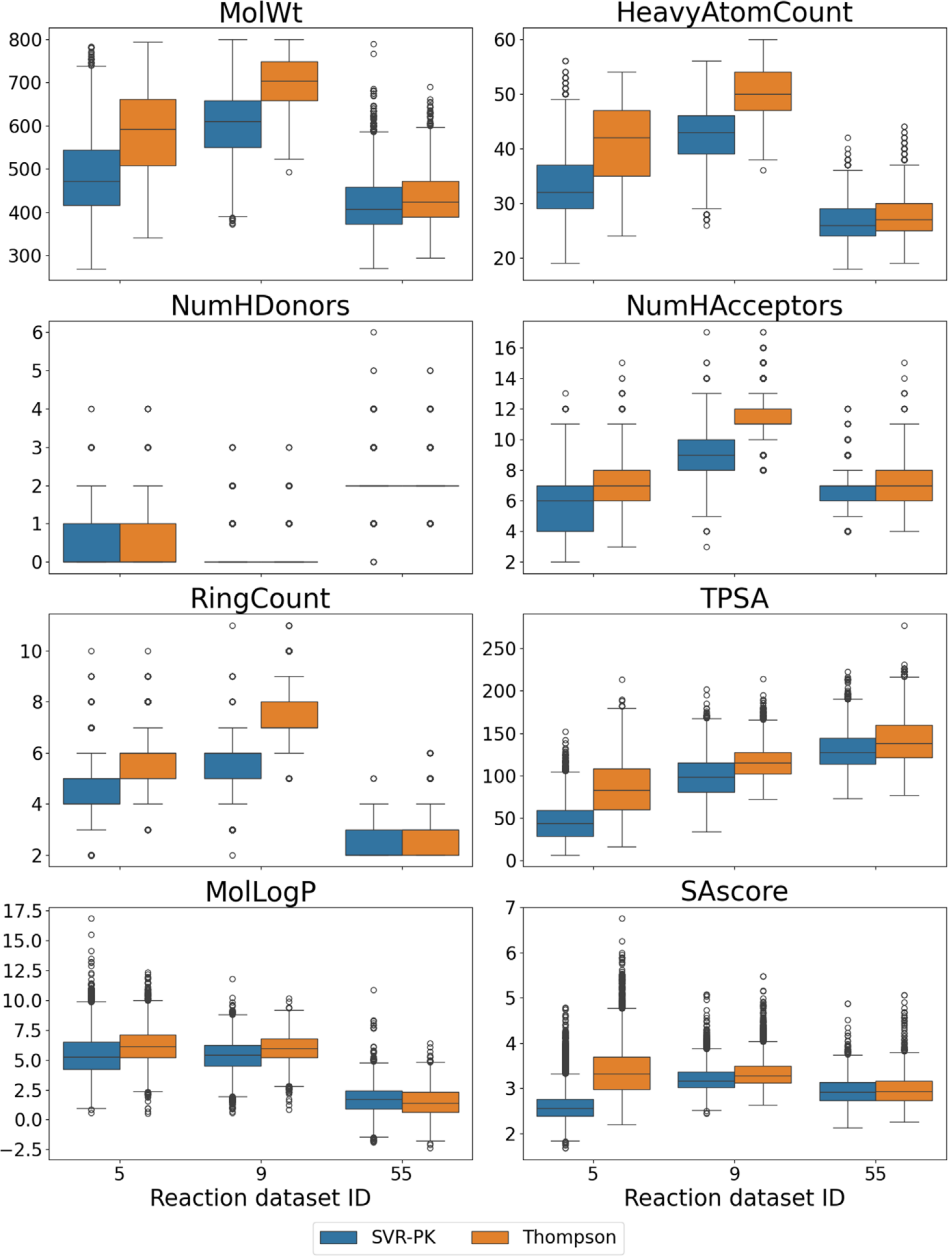
Molecular property distributions for the generated virtual molecules with high potency. The property distributions for SVR‐PK and Thompson sampling are shown in blue and orange, respectively.

Property distributions for the eligible virtual molecules are shown in Figure 6. These properties included molecular weight (MolWt), number of heavy atoms (HeavyAtomCount), number of hydrogen bond donors (NumHDonors), number of hydrogen bond acceptors (NumHAcceptors), number of ring structures (RingCount), topological polar surface area (TPSA), and the synthetic accessibility score (SAscore) [34]. Overall, SVR‐PK generated smaller and simpler molecular structures according to several properties, including MolWt, RingCount, and HeayAtomCounts. SAscore suggested that SVR‐PK‐based molecules were simpler in terms of the molecular structure‐based synthesis accessibility score. The SA score is an index that evaluates the synthetic accessibility of a compound on a scale of 1 (easy) to 10 (difficult).

**FIGURE 7:**
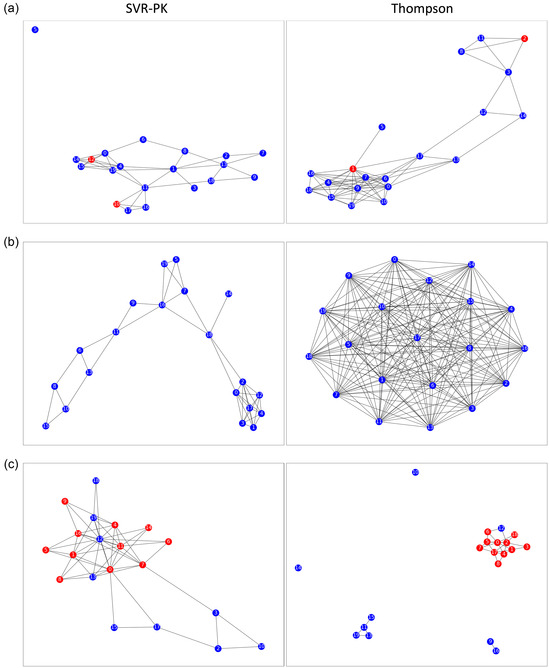
Unique scaffold diversity. A node represents a unique scaffold, and an edge connects scaffolds with a similarity greater than 0.45. Red nodes indicate the common scaffolds for both the SVR‐PK and Thompson sampling approaches, whereas blue nodes indicate scaffolds identified solely by either the SVR‐PK or the Thompson sampling approach. The top 20 scaffolds in terms of the number of occurrences were extracted. Reaction dataset IDs: (a) 5, (b) 9, and (c) 55.

Scaffold diversity was further investigated by analyzing the similarity of the top 20 frequently encountered scaffolds from the two approaches (Figure 7). No clear trend was observed regarding the number of edges (similar scaffolds) between the two approaches. The scaffold‐network densities for ID 5 were 0.080 and 0.067 for the SVR‐PK and Thompson sampling, respectively; those for ID 9 were 0.13 and 0.69; and those for ID 55 were 0.024 and 0.019, respectively.

### 3.4 Scope and Limitations of SVR‐PK‐Based Molecular Generation

The proposed SVR‐PK (SK)‐based molecular generation focuses on a specific reaction scheme, organizing training compounds in a set of reactants per reaction type, enabling the fast evaluation of reactant combinations to produce potentially synthesizable virtual products. Proposed virtual molecules by any structure generation method must be synthesized, and training compounds can be virtually decomposed to reactants. This does not ensure that the training compounds were synthesized using the same reaction scheme. In a situation where training compounds are prepared via different synthesis routes and the same virtual retrosynthesis (decomposition) cannot be identified as that for virtual molecule generation, the SVR‐PK model cannot be created from the training compounds. Our approach restricts training compounds for evaluating virtually synthesizable novel compounds without reducing prediction accuracy through data augmentation. Furthermore, at this stage, only a single reaction step was considered.

**FIGURE 8:**
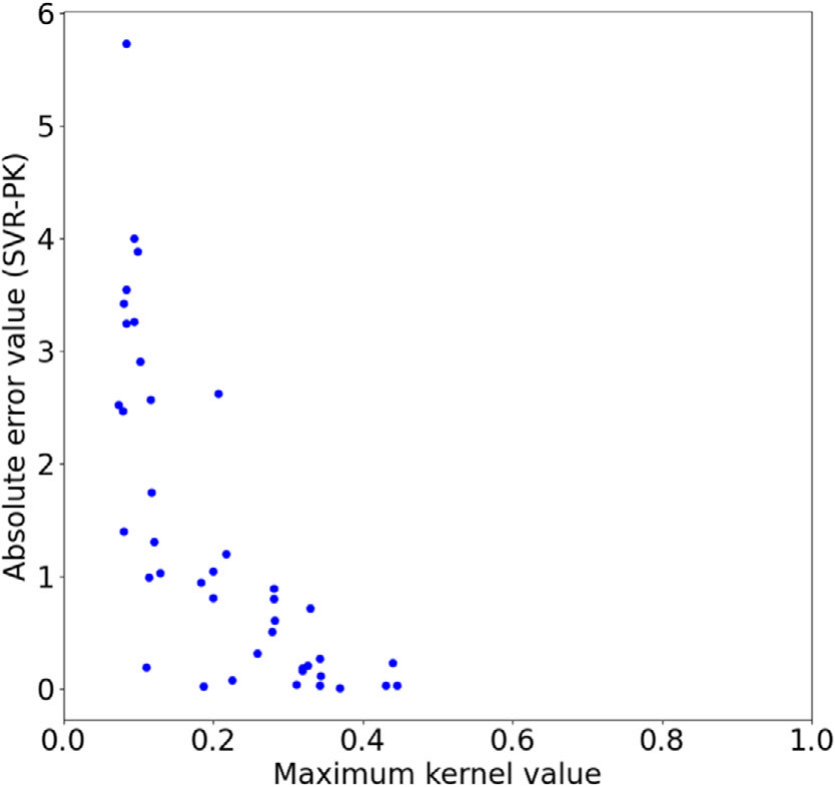
Applicability domain. The maximum kernel values and the absolute prediction error for test compounds (reactant set) for the Reaction dataset ID: 1.

When screening reactants, the domain of applicability of the SVR‐PK model should be considered [35]. In Figure 8, for each test compound (reactants) of the reaction dataset ID 1, the maximum kernel value and the absolute error between the predicted and observed pK_i_ values are plotted. As kernel values (similar to the training data) decreased, the prediction became almost random. To reflect this information into reactant screening, those showing only the maximal kernel values higher than a threshold (for example, 0.2 in Figure 8) can be screened before combining reactants. This reduces the number of candidates and enhances the reliability of the proposed combinations.

## 4 Conclusions

A method for proposing synthesizable novel compounds with desired activity on the basis of an SVM/SVR‐based QSAR model was proposed. The extraction of reactant pairs from a purchasable compound database ensures the synthesizability of the corresponding products through a virtual reaction path. The proposed reactant‐wise kernel functions, in combination with SVM/SVR modeling, enable the fast evaluation of reactant pairs. Owing to a data augmentation strategy through virtually splitting compounds into reactants, the reactant‐based activity prediction models using the proposed modeling method showed comparative prediction accuracy with normal SVR prediction models using ECFP4 as a molecular representation of products (compounds). This trend was confirmed from two types of extrapolation‐oriented datasets across 10 biological targets, with 120 datasets in total. When test datasets consisted of reactant sets different from those in the training dataset, the SVR models using the reactant‐wise kernel function showed no difference in prediction accuracy from those using ECFP4 for products. In the VS demonstration, for one dataset, exhaustive 6.4 × 10^12^ reactant pairs were evaluated by the SVR‐PK model within 8 days using a single computer with an Intel (R) Xeon E5‐2640 v4 @ 2.40 GHz CPU and 128 GB of RAM. The selected virtual molecules were compared with those generated by a Thompson sampling‐based approach, which is scalable for sampling reactant pairs with the desired activity. In two of the three case studies, the Thompson sampling method failed to identify reactant pairs with higher predicted activity found in the SVR‐PK‐based screening, being significantly biased to a few reactants for one of the two reactant components. This highlighted the importance of the exhaustive evaluation of the reactant combinations.

## Author Contributions

**Tomoyuki Miyao** conceived the study and supervised the project. **Yuto Iwasaki** carried out the analysis. **Yuto Iwasaki** and **Tomoyuki Miyao** analyzed the results, participated in the preparation and proofreading of the manuscript and approved the final manuscript.

## Conflicts of Interest

The authors declare no conflicts of interest.

## Supporting information

Supplementary Material

## Data Availability

The codes for curation, model construction, virtual screening of the compounds, and codes for reproducing the figures and tables described in this work are freely available from the Git‐Hub repository at https://github.com/iwmspy/SVR‐PK. The datasets for model construction and screening can be downloaded from the ZENODO repository at https://doi.org/10.5281/zenodo.15525033.

## References

[1] A. Cherkasov , E. N. Muratov , D. Fourches , et al., Journal of Medicinal Chemistry 57, no. 12 (2014): 4977–5010. 24351051 10.1021/jm4004285 PMC4074254

[2] B. J. Neves , R. C. Braga , C. C. Melo‐Filho , J. T. Moreira‐Filho , E. N. Muratov , and C. H. Andrade , Frontiers in Pharmacology 9 (2018): 1275. 30524275 10.3389/fphar.2018.01275 PMC6262347

[3] Y. Bian and X.‐Q. Xie , Cells 11, no. 5 (2022): 915. 35269537 10.3390/cells11050915 PMC8909864

[4] D. Merk , L. Friedrich , F. Grisoni , and G. Schneider , Molecular Informatics 37, no. 1–2 (2018): 1700153. 29319225 10.1002/minf.201700153 PMC5838524

[5] M. H. S. Segler , T. Kogej , C. Tyrchan , and M. P. Waller , ACS Central Science 4, no. 1 (2018): 120–131. 29392184 10.1021/acscentsci.7b00512 PMC5785775

[6] T. Blaschke and J. Bajorath , Journal of Computer‐Aided Molecular Design 36, no. 5 (2022): 363–371. 34046745 10.1007/s10822-021-00392-8 PMC9325839

[7] V. Bagal , R. Aggarwal , P. K. Vinod , and U. D. Priyakumar , Journal of Chemical Information and Modeling 62, no. 9 (2022): 2064–2076. 34694798 10.1021/acs.jcim.1c00600

[8] M. Olivecrona , T. Blaschke , O. Engkvist , and H. Chen , Journal of Cheminformatics 9, no. 1 (2017): 48. 29086083 10.1186/s13321-017-0235-x PMC5583141

[9] J. Lim , S. Ryu , J. W. Kim , and W. Y. Kim , Journal of Cheminformatics 10, no. 1 (2018): 31. 29995272 10.1186/s13321-018-0286-7 PMC6041224

[10] W. Jin , R. Barzilay , and T. Jaakkola , “Junction Tree Variational Autoencoder for Molecular Graph Generation,” Published online March 29, 2019, accessed January 15, 2025, http://arxiv.org/abs/1802.04364.

[11] W. Gao and C. W. Coley , Journal of Chemical Information and Modeling 60, no. 12 (2020): 5714–5723. 32250616 10.1021/acs.jcim.0c00174

[12] D. Mendez , A. Gaulton , A. P. Bento , et al., Nucleic Acids Research 47, no. D1 (2019): D930–D940. 30398643 10.1093/nar/gky1075 PMC6323927

[13] Z. Tu , S. J. Choure , M. H. Fong , et al., “ASKCOS: an open source software suite for synthesilanning,” Published online January 3, 2025, accessed January 15, 2025, http://arxiv.org/abs/2501.01835.

[14] K. Swanson , G. Liu , D. B. Catacutan , A. Arnold , J. Zou , and J. M. Stokes , Nature Machine Intelligence 6, no. 3 (2024): 338–353.

[15] M. Koziarski , A. Rekesh , D. Shevchuk , et al., “RGFN: Synthesizable Molecular Generation Using GFlowNets,” Published online November 6, 2024, accessed January 15, 2025, http://arxiv.org/abs/2406.08506.

[16] W. Gao , R. Mercado , and C. W. Coley , “Amortized Tree Generation for Bottom‐up Synthesis Planning and Synthesizable Molecular Design,” Published online March 12, 2022, accessed January 15, 2025, http://arxiv.org/abs/2110.06389.

[17] J. Bradshaw , B. Paige , M. J. Kusner , M. H. S. Segler , and J. M. Hernández‐Lobato , “A Model to Search for Synthesizable Molecules,” Published online December 4, 2019, accessed January 15, 2025, http://arxiv.org/abs/1906.05221.

[18] S. Seo , J. Lim , and W. Y. Kim , Advanced Science 10, no. 8 (2023): 2206674. 36596675 10.1002/advs.202206674 PMC10015872

[19] R. Rodríguez‐Pérez and J. Bajorath , Journal of Computer‐Aided Molecular Design 36, no. 5 (2022): 355–362. 35304657 10.1007/s10822-022-00442-9 PMC9325859

[20] Y. Shi , Scientific Reports 11, no. 1 (2021): 8806. 33888843 10.1038/s41598-021-88341-1 PMC8062522

[21] M. Singh , J. Rajawat , J. Kuldeep , N. Shukla , D. P. Mishra , and M. I. Siddiqi , Journal of Biomolecular Structure and Dynamics 40, no. 18 (2021): 8494–8507. 33950778 10.1080/07391102.2021.1913229

[22] C.‐A. Azencott , A. Ksikes , S. J. Swamidass , J. H. Chen , L. Ralaivola , and P. Baldi , Journal of Chemical Information and Modeling 47, no. 3 (2007): 965–974. 17338509 10.1021/ci600397p

[23] K. Klarich , B. Goldman , T. Kramer , P. Riley , and W. P. Walters , Journal of Chemical Information and Modeling 64, no. 4 (2024): 1158–1171. 38316125 10.1021/acs.jcim.3c01790 PMC10900287

[24] D. Rogers and M. Hahn , Journal of Chemical Information and Modeling 50, no. 5 (2010): 742–754. 20426451 10.1021/ci100050t

[25] RDKit , “RDKit: Open‐source cheminformatics,” (accessed January 1, 2025), https://www.rdkit.org.

[26] J. Balfer and J. Bajorath , Journal of Chemical Information and Modeling 55, no. 6 (2015): 1136–1147. 25988274 10.1021/acs.jcim.5b00175

[27] R. Rodríguez‐Pérez , M. Vogt , and J. Bajorath , ACS Omega 2, no. 10 (2017): 6371–6379. 30023518 10.1021/acsomega.7b01079 PMC6045367

[28] F. Pedregosa , G. Varoquaux , A. Gramfort , et al., Journal of Machine Learning Research 12 (2011): 2825–2830.

[29] Y. Wang , J. Wang , Z. Cao , and A. B. Farimani , Nature Machine Intelligence 4 (2022): 279–287.

[30] C. W. Coley , L. Rogers , W. H. Green , and K. F. Jensen , ACS Central Science 3, no. 12 (2017): 1237–1245. 29296663 10.1021/acscentsci.7b00355 PMC5746854

[31] eMolecules , “eMolecules: Buy Research Compounds,” (accessed January 1, 2025), https://www.emolecules.com/.

[32] A. F. Zahrt , J. J. Henle , and S. E. Denmark , ACS Combinatorial Science 22, no. 11 (2020): 586–591. 33000621 10.1021/acscombsci.0c00118

[33] J. Götz , M. K. Jackl , C. Jindakun , et al., Science Advances 9, no. 43 (2023): eadj2314. 37889964 10.1126/sciadv.adj2314 PMC10610918

[34] P. Ertl and A. Schuffenhauer , Journal of Cheminformatics 1, no. 1 (2009): 8. 20298526 10.1186/1758-2946-1-8 PMC3225829

[35] W. Klingspohn , M. Mathea , A. Ter Laak , N. Heinrich , and K. Baumann , Journal of Cheminformatics 9, no. 1 (2017): 44. 29086213 10.1186/s13321-017-0230-2 PMC5543028

