# Supplementary material for: Rapid Assessment of Virtually Synthesizable Chemical Structures via Support Vector Machine Models
Source: Mol Inform. 2025 Jul 21;44(7):e202500039. doi: 10.1002/minf.70000 (PMC12278806; doi:10.1002/minf.70000)
Supplement: Supplementary file 1 — Supplementary Material [file MINF-44-e202500039-s001.pdf]

Supporting Information

for

Rapid Assessment of Virtually Synthesizable Chemical Structures via Support Vector Machine Models

Yuto Iwasaki[a] and Tomoyuki Miyao[a], [b]*

[a]Graduate School of Science and Technology, Nara Institute of Science and Technology, 8916-5 Takayama-cho, Ikoma, Nara, 630-0192, Japan.

[b]Data Science Center, Nara Institute of Science and Technology, 8916-5 Takayama-cho, Ikoma, Nara, 630-0192, Japan.

*Corresponding author:

Tomoyuki Miyao; miyao@dsc.naist.jp

Table S1 Reaction types for the reaction datasets

Reaction dataset ID	Count (reactant pairs)	Count (products)	CHEMBL ID	Target	Reaction template
**1**	215	209	214	5-hydroxytryptamine receptor 1A	([CX3+0H0R0:1]-[NX3+0H0R:2])>>[NX3+0H1R:2].[OH]-[CX3+0H0R0:1]
**2**	406	254			([CX4+0H2R0:1]-[NX3+0H1R0:2])>>[NX3+0H2R0:2].[O]=[CX3+0H1R0:1]
**3**	441	283			([cX3+0H0R:1]-[cX3+0H0R:2])>>[Br]-[cX3+0H0R:1].[CH3]-[C]1(-[CH3])-[O]-[B](-[cX3+0H0R:2])-[OX2+0H0R]-[CX4+0H0R]-1(-[CX4+0H3R0])-[CX4+0H3R0]
**4**	515	407			([cX3+0H0R:1]-[OX2+0H0R0:2])>>[F]-[cX3+0H0R:1].[OX2+0H1R0:2]
**5**	2504	2141			([CX4+0H2R0:1]-[NX3+0H0R:2])>>[Cl]-[CX4+0H2R0:1].[NX3+0H1R:2]
**6**	379	318	217	D(2) dopamine receptor	([cX3+0H0R:1]-[OX2+0H0R0:2])>>[F]-[cX3+0H0R:1].[OX2+0H1R0:2]
**7**	3954	3624			([CX4+0H2R0:1]-[NX3+0H0R:2])>>[Br]-[CX4+0H2R0:1].[NX3+0H1R:2]
**8**	157	153	218	Cannabinoid receptor 1	([CX4+0H2R0:1]-[NX3+0H0R:2])>>[Cl]-[CX4+0H2R0:1].[NX3+0H1R:2]
**9**	168	145			([cX3+0H0R:1]-[OX2+0H0R0:2])>>[Cl]-[cX3+0H0R:1].[OX2+0H1R0:2]
**10**	225	206			([cX3+0H0R:1]-[CX3+0H0R0:2])>>[Br]-[cX3+0H0R:1].[CH3]-[O]-[N](-[CH3])-[CX3+0H0R0:2]
**11**	252	233			([CX4+0H2R0:1]-[OX2+0H0R0:2])>>[Br]-[CX4+0H2R0:1].[OX2+0H1R0:2]
**12**	254	248			([CX3+0H0R0:1]-[NX3+0H0R:2])>>[NX3+0H1R:2].[OH]-[CX3+0H0R0:1]
**13**	438	432			([CX4+0H2R0:1]-[nX3+0H0R:2])>>[Br]-[CX4+0H2R0:1].[nX3+0H1R:2]
**14**	1266	1201			([CX3+0H0R0:1]-[NX3+0H1R0:2])>>[NX3+0H2R0:2].[OH]-[CX3+0H0R0:1]
**15**	1416	707			([cX3+0H0R:1]-[cX3+0H0R:2])>>[Br]-[cX3+0H0R:1].[OH]-[B](-[OH])-[cX3+0H0R:2]
**16**	114	114	222	Sodium-dependent noradrenaline transporter	([CX4+0H3R0:1]-[NX3+0H0R:2])>>[NX3+0H1R:2].[O]=[CX3+0H2R0:1]
**17**	140	136			([CX4+0H2R0:1]-[NX3+0H0R:2])>>[Br]-[CX4+0H2R0:1].[NX3+0H1R:2]
**18**	152	146			([CX4+0H3R0:1]-[OX2+0H0R0:2])>>[I]-[CX4+0H3R0:1].[OX2+0H1R0:2]
**19**	165	142			([CX4+0H2R0:1]-[NX3+0H1R0:2])>>[NX3+0H2R0:2].[O]=[CX3+0H1R0:1]
**20**	189	128			([CX4+0H2R0:1]-[NX3+0H0R0:2])>>[Br]-[CX4+0H2R0:1].[NX3+0H1R0:2]
**21**	198	177			([CX4+0H2R0:1]-[OX2+0H0R0:2])>>[Br]-[CX4+0H2R0:1].[OX2+0H1R0:2]
**22**	438	288			([cX3+0H0R:1]-[OX2+0H0R0:2])>>[F]-[cX3+0H0R:1].[OX2+0H1R0:2]
**23**	162	136	224	5-hydroxytryptamine	([CX4+0H3R0:1]-[OX2+0H0R0:2])>>[I]-[CX4+0H3R0:1].[OX2+0H1R0:2]

Reaction dataset ID	Count (reactant pairs)	Count (products)	CHEMBL ID	Target	Reaction template
**24**	233	147		receptor 2A	([cX3+0H0R:1]-[OX2+0H0R0:2])>>[Cl]-[cX3+0H0R:1].[OX2+0H1R0:2]
**25**	393	230			([cX3+0H0R:1]-[cX3+0H0R:2])>>[Br]-[cX3+0H0R:1].[OH]-[B](-[OH])-[cX3+0H0R:2]
**26**	1948	1801			([CX4+0H2R0:1]-[NX3+0H0R:2])>>[Br]-[CX4+0H2R0:1].[NX3+0H1R:2]
**27**	139	122			([CX4+0H3R0:1]-[OX2+0H0R0:2])>>[I]-[CX4+0H3R0:1].[OX2+0H1R0:2]
**28**	167	117			([cX3+0H0R:1]-[OX2+0H0R0:2])>>[Cl]-[cX3+0H0R:1].[OX2+0H1R0:2]
**29**	222	127	225	5-hydroxytryptamine receptor 2C	([cX3+0H0R:1]-[cX3+0H0R:2])>>[Br]-[cX3+0H0R:1].[OH]-[B](-[OH])-[cX3+0H0R:2]
**30**	308	255			([CX3+0H0R0:1]-[NX3+0H1R0:2])>>[NX3+0H2R0:2].[OH]-[CX3+0H0R0:1]
**31**	453	438			([CX4+0H2R0:1]-[NX3+0H0R:2])>>[Br]-[CX4+0H2R0:1].[NX3+0H1R:2]
**32**	144	144			([CX4+0H3R0:1]-[NX3+0H0R:2])>>[NX3+0H1R:2].[O]=[CX3+0H2R0:1]
**33**	184	173			([CX4+0H3R0:1]-[OX2+0H0R0:2])>>[I]-[CX4+0H3R0:1].[OX2+0H1R0:2]
**34**	189	156			([CX4+0H2R0:1]-[NX3+0H1R0:2])>>[NX3+0H2R0:2].[O]=[CX3+0H1R0:1]
**35**	228	158	228	Sodium-dependent serotonin transporter	([CX4+0H2R0:1]-[NX3+0H0R0:2])>>[Br]-[CX4+0H2R0:1].[NX3+0H1R0:2]
**36**	432	365			([CX4+0H2R0:1]-[OX2+0H0R0:2])>>[Br]-[CX4+0H2R0:1].[OX2+0H1R0:2]
**37**	561	394			([cX3+0H0R:1]-[OX2+0H0R0:2])>>[F]-[cX3+0H0R:1].[OX2+0H1R0:2]
**38**	648	610			([CX4+0H2R0:1]-[NX3+0H0R:2])>>[Br]-[CX4+0H2R0:1].[NX3+0H1R:2]
**39**	102	101			([CX3+0H0R0:1]-[cX3+0H0R:2])>>[Cl]-[CX3+0H0R0:1].[cX3+0H1R:2]
**40**	147	144			([CX4+0H3R0:1]-[OX2+0H0R0:2])>>[I]-[CX4+0H3R0:1].[OX2+0H1R0:2]
**41**	155	147	238	Sodium-dependent dopamine transporter	([CX4+0H2R0:1]-[NX3+0H0R:2])>>[Br]-[CX4+0H2R0:1].[NX3+0H1R:2]
**42**	197	175			([CX4+0H2R0:1]-[OX2+0H0R0:2])>>[Br]-[CX4+0H2R0:1].[OX2+0H1R0:2]
**43**	316	219			([cX3+0H0R:1]-[OX2+0H0R0:2])>>[F]-[cX3+0H0R:1].[OX2+0H1R0:2]
**44**	120	119			([CX4+0H2R0:1]-[nX3+0H0R:2])>>[Cl]-[CX4+0H2R0:1].[nX3+0H1R:2]
**45**	131	130			([CX3+0H0R0:1]-[NX3+0H0R0:2])>>[Cl]-[CX3+0H0R0:1].[NX3+0H1R0:2]
**46**	148	126	240	Potassium voltage-gated channel subfamily H member 2	([CX4+0H2R0:1]-[OX2+0H0R0:2])>>[Br]-[CX4+0H2R0:1].[OX2+0H1R0:2]
**47**	163	122			([CX4+0H2R0:1]-[NX3+0H0R0:2])>>[Br]-[CX4+0H2R0:1].[NX3+0H1R0:2]
**48**	172	128			([cX3+0H0R:1]-[OX2+0H0R0:2])>>[F]-[cX3+0H0R:1].[OX2+0H1R0:2]

Reaction dataset ID	Count (reactant pairs)	Count (products)	CHEMBL ID	Target	Reaction template
**49**	202	175			([CX3+0H0R0:1]-[NX3+0H1R0:2])>>[NX3+0H2R0:2].[OH]-[CX3+0H0R0:1]
**50**	257	248			([cX3+0H0R:1]-[NX3+0H0R:2])>>[Cl]-[cX3+0H0R:1].[NX3+0H1R:2]
**51**	361	345			([CX4+0H2R0:1]-[NX3+0H0R:2])>>[Br]-[CX4+0H2R0:1].[NX3+0H1R:2]
**52**	771	369			([cX3+0H0R:1]-[cX3+0H0R:2])>>[Br]-[cX3+0H0R:1].[OH]-[B](-[OH])-[cX3+0H0R:2]
**53**	355	297	261	Carbonic anhydrase 1	([cX3+0H0R:1]-[OX2+0H0R0:2])>>[F]-[cX3+0H0R:1].[OX2+0H1R0:2]
**54**	368	286			([CX4+0H3R0:1]-[OX2+0H0R0:2])>>[I]-[CX4+0H3R0:1].[OX2+0H1R0:2]
**55**	395	341			([CX3+0H0R0:1]-[cX3+0H0R:2])>>[Cl]-[CX3+0H0R0:1].[cX3+0H1R:2]
**56**	399	383			([SX4+0H0R0:1]-[NX3+0H1R0:2])>>[Cl]-[SX4+0H0R0:1].[NX3+0H2R0:2]
**57**	442	411			([CX4+0H2R0:1]-[OX2+0H0R0:2])>>[Br]-[CX4+0H2R0:1].[OX2+0H1R0:2]
**58**	814	549			([cX3+0H0R:1]-[NX3+0H1R0:2])>>[Cl]-[cX3+0H0R:1].[NX3+0H2R0:2]
**59**	1630	785			([cX3+0H0R:1]-[cX3+0H0R:2])>>[Br]-[cX3+0H0R:1].[OH]-[B](-[OH])-[cX3+0H0R:2]
**60**	2193	1640			([CX3+0H0R0:1]-[NX3+0H1R0:2])>>[NX3+0H2R0:2].[OH]-[CX3+0H0R0:1]

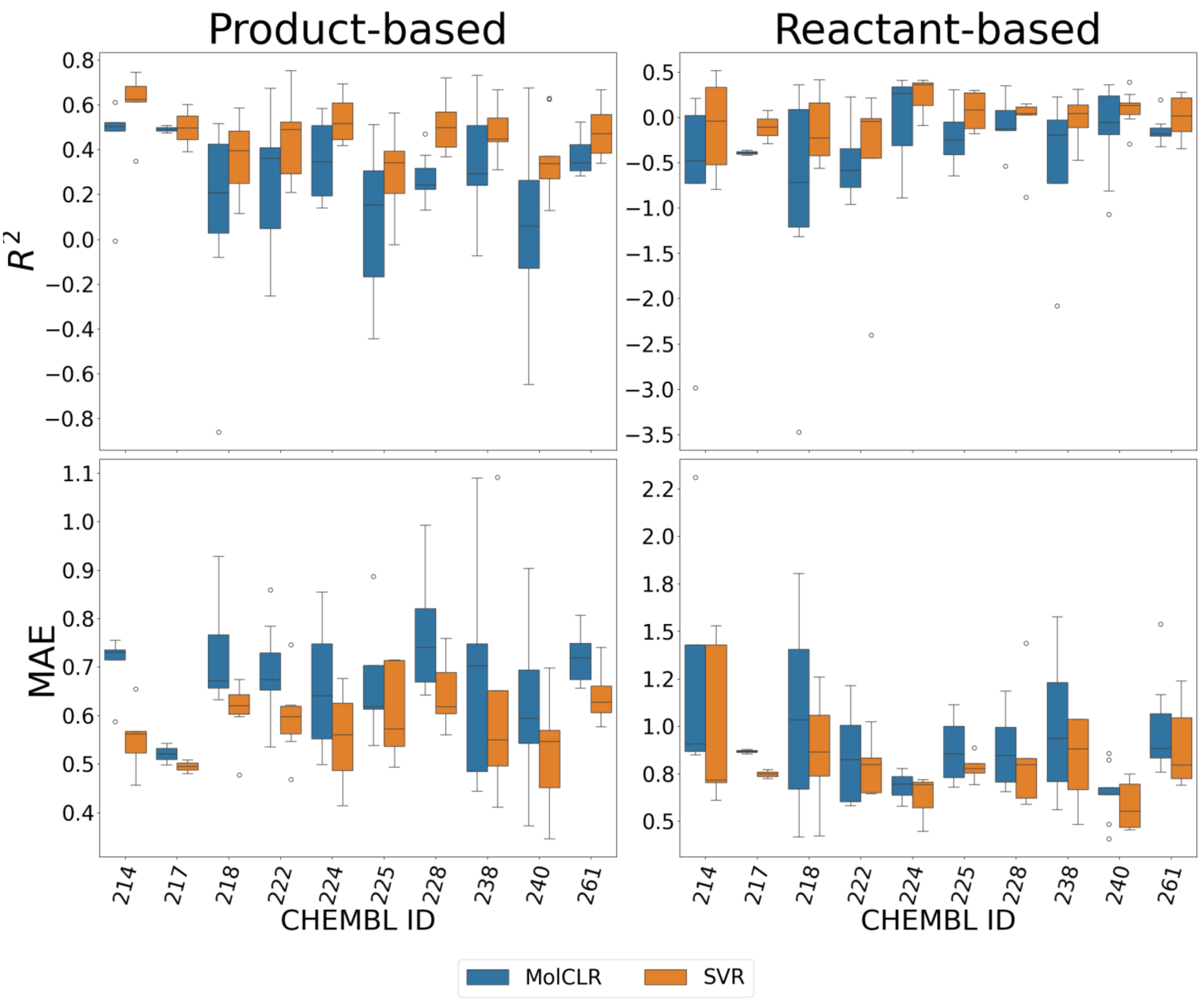

Figure S1 Prediction accuracy of the SVR and MolCLR models. For each target, the prediction accuracy (R2 or MAE) is reported in a box plot. Accuracies for product-based splitting are shown on the left, and those for reactant-based splitting are shown on the right.

Table S2 Prediction accuracies with the best hyperparameters for the training datasets (Product-based without augmentation). (1) SVR-PK, **(2)** SVR-SK, **(3)** SVR-concatECFP, **(4)** SVR-baseline, **(5)** MolCLR.

**Reaction dataset ID**	**Count (reactant pairs)**	**Count (products)**	**R2**					**RMSE**					**MAE**				
			**(1)**	**(2)**	**(3)**	**(4)**	**(5)**	**(1)**	**(2)**	**(3)**	**(4)**	**(5)**	**(1)**	**(2)**	**(3)**	**(4)**	**(5)**
**1**	127	125	0.992	0.964	0.986	0.945	0.770	0.115	0.243	0.150	0.300	0.622	0.074	0.167	0.127	0.253	0.472
**2**	243	152	0.989	0.976	0.985	0.970	0.825	0.157	0.229	0.178	0.257	0.602	0.053	0.078	0.037	0.069	0.495
**3**	257	169	0.779	0.621	0.834	0.684	0.121	0.402	0.526	0.348	0.481	0.787	0.160	0.300	0.133	0.321	0.585
**4**	306	244	0.998	0.981	0.994	0.994	0.891	0.067	0.207	0.118	0.117	0.486	0.008	0.146	0.045	0.019	0.377
**5**	1505	1284	0.994	0.918	0.957	0.950	0.875	0.085	0.326	0.235	0.255	0.403	0.011	0.205	0.075	0.125	0.306
**6**	231	190	0.800	0.882	0.960	0.764	0.573	0.393	0.302	0.176	0.427	0.584	0.189	0.230	0.130	0.194	0.431
**7**	2389	2174	0.995	0.896	0.970	0.988	0.917	0.072	0.330	0.176	0.112	0.293	0.010	0.208	0.064	0.072	0.222
**8**	94	91	1.000	1.000	1.000	1.000	0.719	0.001	0.005	0.002	0.024	0.602	0.001	0.002	0.002	0.004	0.480
**9**	100	87	0.951	0.974	0.976	0.922	-0.575	0.229	0.166	0.161	0.287	1.279	0.105	0.134	0.132	0.147	1.051
**10**	136	123	0.985	0.908	0.850	0.987	0.441	0.117	0.290	0.370	0.109	0.730	0.025	0.148	0.169	0.045	0.596
**11**	148	139	0.872	0.621	0.835	0.809	0.199	0.336	0.577	0.381	0.410	0.859	0.207	0.353	0.245	0.318	0.680
**12**	152	148	0.990	0.890	0.918	0.970	0.553	0.103	0.336	0.290	0.174	0.673	0.067	0.153	0.155	0.085	0.543
**13**	261	259	0.999	0.931	0.983	0.978	0.178	0.034	0.272	0.134	0.153	0.952	0.005	0.129	0.122	0.124	0.757
**14**	762	720	0.960	0.858	0.918	0.920	0.719	0.221	0.415	0.315	0.311	0.586	0.099	0.323	0.216	0.213	0.465
**15**	854	424	0.994	0.963	0.989	0.990	0.706	0.087	0.224	0.120	0.117	0.625	0.022	0.152	0.038	0.038	0.476
**16**	68	68	0.993	0.993	0.991	0.992	0.710	0.087	0.087	0.096	0.090	0.556	0.014	0.014	0.018	0.015	0.426
**17**	83	81	0.993	0.947	0.974	0.943	0.795	0.061	0.168	0.116	0.174	0.321	0.060	0.134	0.113	0.069	0.258
**18**	90	87	0.987	0.987	0.985	0.992	0.939	0.188	0.188	0.204	0.148	0.398	0.060	0.060	0.066	0.134	0.309
**19**	99	85	0.999	0.976	0.997	0.997	0.863	0.042	0.183	0.067	0.067	0.406	0.034	0.137	0.062	0.063	0.338
**20**	117	76	1.000	1.000	1.000	0.943	0.633	0.001	0.014	0.001	0.238	0.596	0.001	0.003	0.001	0.138	0.453
**21**	118	106	0.962	0.955	0.962	0.899	0.817	0.228	0.251	0.228	0.374	0.514	0.213	0.223	0.212	0.225	0.403
**22**	261	172	0.986	0.932	0.988	0.986	0.857	0.130	0.292	0.125	0.132	0.429	0.119	0.246	0.060	0.057	0.322

Reaction dataset ID	Count (reactant pairs)	Count (products)	R2 (1)	R2 (2)	R2 (3)	R2 (4)	R2 (5)	RMSE (1)	RMSE (2)	RMSE (3)	RMSE (4)	RMSE (5)	MAE (1)	MAE (2)	MAE (3)	MAE (4)	MAE (5)
**23**	98	81	1.000	1.000	1.000	0.835	0.834	0.001	0.022	0.001	0.487	0.471	0.001	0.005	0.001	0.198	0.359
**24**	141	88	0.998	0.930	0.985	0.985	0.913	0.044	0.260	0.119	0.120	0.301	0.034	0.224	0.116	0.118	0.232
**25**	237	138	0.998	0.962	0.992	0.994	0.755	0.051	0.236	0.110	0.093	0.606	0.007	0.215	0.073	0.018	0.474
**26**	1168	1080	0.976	0.925	0.968	0.928	0.874	0.166	0.296	0.193	0.290	0.385	0.066	0.185	0.098	0.149	0.286
**27**	85	73	0.978	0.977	0.976	0.920	0.803	0.120	0.122	0.125	0.227	0.369	0.117	0.119	0.120	0.217	0.300
**28**	101	70	0.963	0.961	0.950	0.999	0.823	0.205	0.211	0.238	0.026	0.448	0.042	0.049	0.060	0.005	0.321
**29**	133	76	0.820	0.770	0.874	0.759	-0.271	0.333	0.378	0.280	0.386	0.880	0.223	0.298	0.154	0.260	0.709
**30**	188	153	0.999	0.952	0.977	0.932	0.491	0.034	0.193	0.134	0.229	0.636	0.004	0.065	0.022	0.092	0.503
**31**	269	262	0.999	0.966	0.994	0.995	0.930	0.029	0.194	0.084	0.072	0.276	0.004	0.145	0.020	0.030	0.215
**32**	86	86	0.997	0.997	0.996	0.997	0.867	0.067	0.067	0.073	0.069	0.444	0.009	0.009	0.011	0.010	0.373
**33**	113	103	0.999	0.997	0.999	0.999	0.956	0.066	0.124	0.069	0.068	0.462	0.008	0.035	0.009	0.009	0.383
**34**	111	93	0.932	0.844	0.912	0.972	0.893	0.360	0.542	0.408	0.230	0.457	0.119	0.333	0.281	0.222	0.378
**35**	138	94	0.990	0.843	0.918	0.921	0.697	0.132	0.514	0.373	0.365	0.708	0.029	0.215	0.136	0.124	0.536
**36**	266	219	0.943	0.951	0.989	0.984	0.783	0.285	0.264	0.124	0.150	0.563	0.076	0.230	0.117	0.086	0.446
**37**	325	236	1.000	0.959	0.998	0.997	0.888	0.023	0.228	0.052	0.060	0.380	0.003	0.204	0.006	0.008	0.286
**38**	392	366	0.971	0.881	0.915	0.967	0.743	0.171	0.349	0.296	0.183	0.512	0.052	0.161	0.159	0.072	0.433
**39**	60	60	0.773	0.530	0.677	0.553	0.673	0.376	0.541	0.449	0.528	0.445	0.200	0.413	0.285	0.403	0.335
**40**	89	86	0.995	0.927	0.993	0.993	0.957	0.270	1.001	0.315	0.314	0.684	0.161	0.420	0.178	0.122	0.538
**41**	93	88	1.000	0.962	0.989	0.997	0.937	0.001	0.218	0.117	0.059	0.282	0.001	0.206	0.113	0.058	0.221
**42**	115	105	0.993	0.989	0.985	1.000	0.831	0.091	0.113	0.133	0.008	0.460	0.021	0.081	0.063	0.002	0.331
**43**	194	131	0.848	0.880	0.935	0.932	0.839	0.377	0.335	0.247	0.252	0.402	0.165	0.151	0.154	0.088	0.308
**44**	72	71	1.000	0.985	0.978	0.923	0.660	0.001	0.094	0.114	0.211	0.448	0.001	0.047	0.109	0.192	0.348
**45**	79	78	0.901	0.901	0.901	0.903	0.698	0.212	0.212	0.211	0.210	0.371	0.196	0.196	0.195	0.193	0.296
**46**	90	75	1.000	0.918	0.997	1.000	0.452	0.001	0.304	0.062	0.001	0.759	0.001	0.247	0.008	0.001	0.632
**47**	94	73	1.000	0.989	1.000	0.998	0.878	0.001	0.082	0.001	0.031	0.270	0.001	0.065	0.001	0.030	0.209
**48**	101	76	0.737	0.829	0.890	0.711	0.042	0.492	0.397	0.319	0.516	0.978	0.307	0.217	0.120	0.327	0.670

**Reaction dataset ID**	**Count (reactant pairs)**	**Count (products)**	**R2**					**RMSE**					**MAE**				
			**(1)**	**(2)**	**(3)**	**(4)**	**(5)**	**(1)**	**(2)**	**(3)**	**(4)**	**(5)**	**(1)**	**(2)**	**(3)**	**(4)**	**(5)**
**49**	124	105	1.000	0.977	1.000	0.924	0.054	0.008	0.124	0.001	0.225	0.789	0.008	0.121	0.001	0.214	0.629
**50**	155	148	0.994	0.995	0.982	0.998	0.874	0.070	0.065	0.119	0.035	0.320	0.064	0.040	0.112	0.008	0.254
**51**	215	207	0.993	0.743	0.794	0.977	0.899	0.064	0.395	0.353	0.117	0.252	0.061	0.240	0.229	0.114	0.197
**52**	458	221	0.999	0.915	0.989	0.959	0.602	0.036	0.274	0.097	0.190	0.612	0.004	0.135	0.043	0.098	0.472
**53**	206	178	0.992	0.978	0.982	0.973	0.857	0.100	0.160	0.144	0.177	0.409	0.016	0.036	0.030	0.135	0.311
**54**	221	171	0.988	0.988	0.986	0.985	0.912	0.164	0.164	0.176	0.180	0.448	0.029	0.029	0.033	0.039	0.345
**55**	235	204	0.998	0.975	0.999	0.991	0.829	0.049	0.172	0.039	0.102	0.452	0.006	0.136	0.005	0.017	0.352
**56**	235	229	0.920	0.880	0.890	0.902	0.915	0.405	0.495	0.474	0.447	0.392	0.031	0.198	0.071	0.063	0.300
**57**	261	246	0.915	0.783	0.805	0.857	0.791	0.357	0.569	0.539	0.462	0.549	0.252	0.453	0.436	0.271	0.392
**58**	476	329	0.987	0.909	0.930	0.932	0.682	0.142	0.372	0.327	0.322	0.706	0.009	0.241	0.077	0.024	0.477
**59**	981	471	0.999	0.904	0.950	0.948	0.863	0.035	0.344	0.247	0.251	0.413	0.003	0.187	0.213	0.216	0.302
**60**	1312	984	0.996	0.900	0.982	0.985	0.883	0.092	0.465	0.195	0.181	0.480	0.063	0.322	0.134	0.133	0.362

Table S3 Prediction accuracies with the best hyperparameters for the training datasets (Product-based with augmentation). (1) SVR-PK, **(2)** SVR-SK, **(3)** SVR-concatECFP. SVR-baseline and MolCLR results are the same as in **Table S2** and therefore omitted.

**Reaction dataset ID**	**Count (reactant pairs)**	**R2**			**RMSE**			**MAE**		
		**(1)**	**(2)**	**(3)**	**(1)**	**(2)**	**(3)**	**(1)**	**(2)**	**(3)**
**2**	248	0.989	0.972	0.984	0.156	0.248	0.184	0.039	0.156	0.089
**3**	342	0.990	0.746	0.837	0.086	0.430	0.345	0.009	0.256	0.151
**4**	321	0.994	0.982	0.980	0.112	0.202	0.210	0.018	0.106	0.049
**5**	1518	0.994	0.919	0.980	0.085	0.324	0.161	0.011	0.176	0.083
**6**	239	0.924	0.912	0.887	0.243	0.261	0.296	0.069	0.155	0.172
**7**	2411	0.995	0.896	0.970	0.072	0.330	0.176	0.010	0.207	0.056
**9**	102	0.991	0.974	0.984	0.096	0.166	0.130	0.015	0.136	0.079
**10**	141	0.985	0.905	0.846	0.118	0.294	0.374	0.026	0.107	0.168
**11**	150	0.879	0.743	0.834	0.326	0.475	0.381	0.126	0.357	0.245
**12**	153	0.953	0.797	0.915	0.220	0.456	0.295	0.151	0.400	0.193
**13**	262	0.962	0.930	0.900	0.201	0.274	0.327	0.065	0.120	0.150
**14**	769	0.982	0.904	0.962	0.149	0.341	0.216	0.084	0.276	0.156
**15**	946	0.983	0.920	0.987	0.150	0.328	0.134	0.035	0.143	0.041
**22**	276	0.997	0.953	0.994	0.057	0.243	0.087	0.008	0.218	0.067
**23**	101	1.000	1.000	1.000	0.016	0.025	0.015	0.015	0.018	0.015
**24**	158	0.996	0.959	0.996	0.060	0.198	0.061	0.059	0.135	0.059
**25**	282	0.996	0.984	0.990	0.074	0.151	0.121	0.010	0.089	0.029
**26**	1174	0.975	0.889	0.964	0.169	0.359	0.206	0.089	0.223	0.137
**27**	88	0.978	0.977	0.977	0.121	0.123	0.121	0.119	0.120	0.119
**28**	116	1.000	0.961	1.000	0.001	0.211	0.001	0.001	0.050	0.001
**29**	152	0.817	0.793	0.724	0.337	0.358	0.413	0.229	0.239	0.280
**31**	271	0.999	0.970	0.994	0.029	0.183	0.084	0.004	0.063	0.020
**36**	270	0.943	0.971	0.998	0.285	0.202	0.058	0.076	0.142	0.008

**Reaction dataset ID**	**Count (reactant pairs)**	**R2**			**RMSE**			**MAE**		
		**(1)**	**(2)**	**(3)**	**(1)**	**(2)**	**(3)**	**(1)**	**(2)**	**(3)**
**37**	356	1.000	0.958	0.998	0.022	0.231	0.055	0.003	0.204	0.006
**38**	393	0.966	0.931	0.904	0.186	0.266	0.314	0.054	0.167	0.194
**42**	118	1.000	0.988	1.000	0.001	0.118	0.015	0.001	0.114	0.015
**43**	204	0.964	0.878	0.987	0.182	0.338	0.110	0.046	0.151	0.071
**46**	113	1.000	0.991	0.996	0.001	0.102	0.066	0.001	0.022	0.009
**47**	103	1.000	0.989	1.000	0.001	0.080	0.001	0.001	0.064	0.001
**48**	102	0.737	0.830	0.887	0.493	0.395	0.322	0.307	0.181	0.100
**49**	125	1.000	0.977	1.000	0.015	0.124	0.008	0.015	0.121	0.008
**52**	502	0.980	0.913	0.951	0.133	0.276	0.208	0.026	0.135	0.104
**53**	215	0.997	0.978	0.982	0.056	0.161	0.146	0.005	0.037	0.031
**54**	226	0.996	0.993	0.996	0.098	0.128	0.099	0.011	0.023	0.011
**55**	248	1.000	0.975	0.999	0.001	0.171	0.038	0.001	0.135	0.004
**56**	236	0.920	0.843	0.890	0.405	0.567	0.474	0.031	0.230	0.070
**57**	264	0.947	0.837	0.880	0.282	0.493	0.423	0.236	0.433	0.271
**58**	494	0.987	0.910	0.925	0.143	0.370	0.338	0.009	0.240	0.130
**59**	1077	0.996	0.892	0.947	0.068	0.363	0.256	0.004	0.197	0.213
**60**	1352	0.992	0.899	0.982	0.135	0.466	0.196	0.117	0.387	0.134

Table S4 Prediction accuracies with the best hyperparameters for the training datasets (Reactant-based without augmentation). (1) SVR-PK, **(2)** SVR-SK, **(3)** SVR-concatECFP, **(4)** SVR-baseline, **(5)** MolCLR. Blank cells in the MolCLR results mean that the evaluation could not be performed on that reaction data set.

Reaction dataset ID	Count (reactant pairs)	Count (products)	R2					RMSE					MAE				
			(1)	(2)	(3)	(4)	(5)	(1)	(2)	(3)	(4)	(5)	(1)	(2)	(3)	(4)	(5)
**1**	51	51	0.987	0.934	0.958	0.960	0.610	0.118	0.270	0.216	0.209	0.671	0.038	0.122	0.092	0.095	0.576
**2**	98	95	0.999	0.980	0.976	0.991	0.735	0.038	0.206	0.228	0.142	0.753	0.005	0.146	0.154	0.128	0.583
**3**	86	83	0.767	0.819	0.657	0.727	0.635	0.443	0.390	0.538	0.480	0.573	0.213	0.189	0.351	0.269	0.412
**4**	108	105	0.995	0.987	0.992	0.967	0.724	0.098	0.151	0.119	0.239	0.682	0.014	0.080	0.032	0.214	0.494
**5**	424	422	0.999	0.982	0.998	0.998	0.816	0.044	0.155	0.047	0.047	0.491	0.006	0.128	0.021	0.007	0.378
**6**	88	85	1.000	0.723	0.995	0.974	0.598	0.017	0.501	0.066	0.155	0.553	0.003	0.242	0.061	0.130	0.447
**7**	656	653	0.997	0.946	0.979	0.975	0.906	0.064	0.263	0.162	0.178	0.347	0.010	0.164	0.089	0.134	0.276
**8**	31	28	1.000	0.873	0.996	1.000	-1.234	0.001	0.356	0.060	0.002	1.505	0.001	0.237	0.059	0.002	1.287
**9**	33	29	0.295	0.807	0.793	0.637	-1.409	0.791	0.414	0.429	0.567	1.534	0.559	0.388	0.407	0.339	1.089
**10**	52	52	0.994	0.686	0.949	0.979	-0.330	0.088	0.627	0.252	0.164	1.300	0.018	0.363	0.230	0.131	0.979
**11**	63	63	0.798	0.789	0.934	0.934	-0.183	0.416	0.426	0.237	0.238	0.988	0.258	0.179	0.222	0.222	0.728
**12**	70	70	0.839	0.852	0.788	0.799	-0.258	0.372	0.357	0.427	0.415	1.022	0.182	0.165	0.218	0.211	0.856
**13**	168	168	0.960	0.950	0.966	0.968	0.539	0.198	0.222	0.184	0.178	0.674	0.142	0.150	0.137	0.135	0.522
**14**	339	336	0.946	0.945	0.938	0.951	0.606	0.261	0.263	0.278	0.247	0.681	0.234	0.233	0.240	0.226	0.543
**15**	344	316	0.998	0.984	0.997	0.996	0.923	0.049	0.146	0.059	0.071	0.314	0.006	0.126	0.022	0.023	0.250
**16**	63	63	1.000	0.998	1.000	1.000		0.001	0.039	0.001	0.001		0.001	0.009	0.001	0.001	
**17**	39	38	0.293	0.637	0.651	0.157	0.517	0.700	0.501	0.492	0.764	0.592	0.617	0.447	0.442	0.564	0.482
**18**	84	82	0.993	0.983	0.981	0.996		0.140	0.217	0.232	0.111		0.126	0.128	0.221	0.074	
**19**	73	73	0.991	0.994	0.991	0.964	0.825	0.121	0.099	0.121	0.239	0.528	0.119	0.038	0.118	0.219	0.438
**20**	46	46	0.827	0.829	0.826	0.824	0.884	0.428	0.425	0.429	0.431	0.365	0.391	0.390	0.394	0.398	0.305
**21**	48	48	0.452	0.521	0.722	0.448	0.532	0.750	0.702	0.534	0.753	0.677	0.527	0.486	0.452	0.538	0.492

Reaction dataset ID	Count (reactant pairs)	Count (products)	R2					RMSE					MAE				
			(1)	(2)	(3)	(4)	(5)	(1)	(2)	(3)	(4)	(5)	(1)	(2)	(3)	(4)	(5)
**22**	77	74	0.960	0.837	0.882	0.926	0.599	0.176	0.354	0.302	0.238	0.538	0.045	0.213	0.185	0.220	0.421
**23**	89	69	0.997	0.911	0.997	0.751		0.061	0.332	0.061	0.556		0.060	0.253	0.061	0.296	
**24**	48	43	1.000	1.000	1.000	1.000	0.570	0.001	0.001	0.015	0.001	0.565	0.001	0.001	0.003	0.001	0.492
**25**	101	100	0.992	0.989	0.994	0.990	0.686	0.109	0.125	0.097	0.118	0.680	0.017	0.020	0.011	0.018	0.535
**26**	314	310	0.983	0.940	0.950	0.949	0.854	0.161	0.302	0.277	0.278	0.469	0.043	0.167	0.181	0.131	0.395
**27**	76	72	0.672	0.672	0.654	0.640		0.429	0.429	0.440	0.449		0.158	0.158	0.177	0.227	
**28**	35	34	1.000	0.939	0.961	0.964	0.825	0.001	0.336	0.270	0.257	0.555	0.001	0.189	0.110	0.182	0.440
**29**	63	62	0.671	0.584	0.629	0.630	0.359	0.448	0.504	0.476	0.476	0.632	0.395	0.422	0.410	0.321	0.498
**30**	73	73	0.924	0.912	0.873	0.869	0.441	0.264	0.284	0.342	0.347	0.711	0.055	0.064	0.110	0.128	0.553
**31**	112	109	0.980	0.927	1.000	0.948	0.789	0.109	0.210	0.008	0.177	0.357	0.017	0.095	0.008	0.096	0.273
**32**	79	79	0.988	0.985	0.988	0.988		0.120	0.132	0.120	0.120		0.118	0.123	0.117	0.117	
**33**	101	96	0.997	0.995	0.997	0.999		0.130	0.160	0.131	0.085		0.125	0.137	0.125	0.070	
**34**	83	80	0.990	0.988	0.987	0.990	0.913	0.118	0.128	0.133	0.116	0.352	0.114	0.117	0.120	0.112	0.285
**35**	52	52	0.999	0.970	0.990	0.991	0.906	0.031	0.223	0.131	0.124	0.328	0.031	0.212	0.124	0.119	0.257
**36**	109	108	0.982	0.905	0.966	0.884	-0.005	0.152	0.345	0.206	0.382	1.158	0.045	0.257	0.133	0.276	0.946
**37**	110	109	0.945	0.885	0.882	0.850	0.511	0.278	0.401	0.407	0.458	0.776	0.111	0.161	0.274	0.285	0.668
**38**	140	136	0.968	0.762	0.983	0.977	0.885	0.225	0.610	0.163	0.191	0.416	0.119	0.438	0.087	0.134	0.326
**39**	26	26	0.901	0.889	0.882	0.882	0.291	0.265	0.281	0.290	0.290	0.714	0.078	0.129	0.105	0.077	0.520
**40**	81	81	0.991	0.936	0.986	0.982		0.371	0.966	0.458	0.506		0.195	0.537	0.171	0.322	
**41**	44	41	1.000	1.000	1.000	0.999	0.907	0.001	0.016	0.001	0.031	0.378	0.001	0.003	0.001	0.031	0.274
**42**	48	48	0.841	0.719	0.820	0.669	-0.029	0.376	0.499	0.399	0.542	0.936	0.177	0.277	0.195	0.336	0.700
**43**	56	55	0.486	0.391	0.421	0.409	0.782	0.425	0.462	0.451	0.455	0.289	0.128	0.183	0.164	0.161	0.224
**44**	33	33	1.000	1.000	1.000	1.000	-0.016	0.002	0.001	0.001	0.001	0.882	0.002	0.001	0.001	0.001	0.738
**45**	57	57	0.913	0.913	0.948	0.951	0.797	0.184	0.184	0.142	0.137	0.269	0.072	0.064	0.091	0.089	0.215
**46**	45	43	0.777	0.598	0.638	0.314	-0.370	0.490	0.657	0.624	0.859	1.229	0.344	0.529	0.518	0.599	1.014

Reaction dataset ID	Count (reactant pairs)	Count (products)	R2					RMSE					MAE				
			(1)	(2)	(3)	(4)	(5)	(1)	(2)	(3)	(4)	(5)	(1)	(2)	(3)	(4)	(5)
**47**	49	36	0.946	1.000	1.000	0.900	0.151	0.189	0.001	0.001	0.257	0.791	0.070	0.001	0.001	0.117	0.656
**48**	51	48	0.773	0.560	0.719	0.748	0.484	0.448	0.625	0.499	0.472	0.679	0.197	0.401	0.273	0.264	0.532
**49**	70	67	0.665	0.541	0.602	0.765	0.543	0.452	0.529	0.493	0.379	0.532	0.258	0.386	0.329	0.230	0.410
**50**	97	97	1.000	0.999	0.999	1.000	0.944	0.001	0.030	0.024	0.014	0.213	0.001	0.005	0.004	0.005	0.167
**51**	90	89	1.000	1.000	1.000	1.000	0.808	0.001	0.001	0.001	0.001	0.304	0.001	0.001	0.001	0.001	0.236
**52**	209	184	0.994	0.937	0.980	0.721	-0.337	0.061	0.194	0.109	0.408	0.902	0.059	0.140	0.074	0.293	0.672
**53**	70	70	0.907	0.649	0.852	0.876	-0.363	0.299	0.581	0.377	0.345	1.144	0.087	0.259	0.157	0.131	0.874
**54**	203	168	0.993	0.993	0.991	0.988		0.111	0.111	0.128	0.152		0.015	0.015	0.018	0.024	
**55**	78	76	0.997	1.000	1.000	1.000	0.651	0.070	0.001	0.001	0.001	0.754	0.009	0.001	0.001	0.001	0.581
**56**	89	88	1.000	0.989	1.000	1.000	0.895	0.002	0.131	0.002	0.002	0.396	0.001	0.034	0.001	0.001	0.320
**57**	114	113	0.972	0.979	0.968	0.973	0.708	0.235	0.201	0.252	0.231	0.780	0.225	0.131	0.144	0.065	0.601
**58**	243	235	0.891	0.577	0.742	0.844	0.490	0.371	0.729	0.570	0.442	0.803	0.026	0.339	0.181	0.143	0.508
**59**	320	304	0.993	0.979	0.991	0.983	0.410	0.083	0.141	0.094	0.128	0.749	0.017	0.121	0.067	0.115	0.569
**60**	496	490	0.783	0.753	0.704	0.717	0.298	0.542	0.579	0.634	0.619	0.983	0.218	0.340	0.380	0.337	0.699

Table S5 Prediction accuracies with the best hyperparameters for the training datasets (Reactant-based with augmentation). (1) SVR-PK, **(2)** SVR-SK, **(3)** SVR-concatECFP. SVR-baseline and MolCLR results are the same as in **Table S4** and therefore omitted. Only the prediction accuracies of the reaction datasets for which data augmentation could be performed are reported.

**Reaction dataset ID**	**Count (reactant pairs)**	**R2**			**RMSE**			**MAE**		
		**(1)**	**(2)**	**(3)**	**(1)**	**(2)**	**(3)**	**(1)**	**(2)**	**(3)**
**1**	54	0.987	0.940	0.962	0.118	0.258	0.206	0.038	0.148	0.124
**2**	161	1.000	0.999	0.998	0.001	0.048	0.069	0.001	0.035	0.009
**3**	166	0.778	0.623	0.682	0.432	0.563	0.518	0.169	0.327	0.234
**4**	156	0.995	0.988	0.981	0.097	0.143	0.180	0.014	0.052	0.041
**5**	523	0.998	0.989	0.998	0.045	0.122	0.046	0.006	0.054	0.007
**6**	109	0.998	0.932	0.948	0.037	0.248	0.217	0.031	0.224	0.205
**7**	783	1.000	0.888	0.973	0.024	0.378	0.184	0.002	0.177	0.134
**9**	36	0.806	0.856	0.868	0.415	0.357	0.342	0.211	0.221	0.248
**10**	66	0.995	0.887	0.981	0.080	0.377	0.153	0.017	0.245	0.088
**11**	69	0.990	0.973	0.989	0.094	0.153	0.097	0.072	0.132	0.073
**12**	73	0.847	0.866	0.797	0.362	0.339	0.418	0.180	0.163	0.216
**13**	169	0.960	0.950	0.966	0.198	0.222	0.184	0.141	0.149	0.137
**14**	342	0.946	0.945	0.939	0.262	0.263	0.278	0.235	0.232	0.239
**15**	728	0.998	0.985	0.995	0.046	0.143	0.078	0.006	0.125	0.064
**17**	41	0.197	0.167	0.176	0.746	0.759	0.755	0.561	0.565	0.560
**19**	78	0.851	0.968	0.838	0.484	0.225	0.504	0.269	0.214	0.282
**20**	79	1.000	1.000	1.000	0.001	0.001	0.002	0.001	0.001	0.002
**21**	58	0.467	0.736	0.451	0.740	0.521	0.751	0.472	0.449	0.493
**22**	140	0.961	0.825	0.901	0.172	0.367	0.276	0.067	0.271	0.136
**23**	92	1.000	0.999	1.000	0.001	0.035	0.001	0.001	0.007	0.001
**24**	77	1.000	1.000	1.000	0.001	0.001	0.001	0.001	0.001	0.001
**25**	200	0.992	0.990	0.986	0.106	0.122	0.145	0.016	0.019	0.025

**Reaction dataset ID**	**Count (reactant pairs)**	**R2**			**RMSE**			**MAE**		
		**(1)**	**(2)**	**(3)**	**(1)**	**(2)**	**(3)**	**(1)**	**(2)**	**(3)**
**26**	343	0.982	0.939	0.952	0.163	0.305	0.269	0.043	0.199	0.144
**27**	80	0.848	0.848	0.830	0.291	0.291	0.308	0.062	0.062	0.067
**28**	51	1.000	0.985	0.997	0.001	0.169	0.078	0.001	0.058	0.022
**29**	124	0.789	0.667	0.585	0.359	0.451	0.504	0.291	0.267	0.368
**30**	102	0.926	0.914	0.872	0.262	0.282	0.343	0.053	0.065	0.112
**31**	117	0.980	0.920	0.954	0.111	0.220	0.168	0.018	0.087	0.074
**34**	87	0.950	0.949	0.944	0.260	0.262	0.274	0.227	0.226	0.237
**35**	91	1.000	0.873	1.000	0.001	0.456	0.001	0.001	0.197	0.001
**36**	133	0.982	0.945	0.939	0.150	0.264	0.276	0.017	0.232	0.234
**37**	200	0.941	0.861	0.882	0.287	0.443	0.408	0.065	0.179	0.137
**38**	151	0.964	0.888	0.921	0.236	0.419	0.352	0.071	0.201	0.144
**39**	27	0.901	0.800	0.795	0.266	0.377	0.382	0.078	0.211	0.213
**41**	46	1.000	1.000	1.000	0.001	0.019	0.001	0.001	0.004	0.001
**42**	61	0.992	0.730	0.812	0.085	0.490	0.408	0.045	0.268	0.200
**43**	109	0.123	0.096	0.101	0.555	0.563	0.561	0.309	0.320	0.318
**46**	59	0.779	0.821	0.888	0.487	0.439	0.348	0.290	0.319	0.204
**47**	75	0.965	0.961	1.000	0.153	0.160	0.001	0.052	0.060	0.001
**48**	69	0.765	0.553	0.576	0.456	0.630	0.613	0.237	0.405	0.392
**49**	80	0.848	0.729	0.769	0.304	0.407	0.376	0.136	0.272	0.253
**50**	100	1.000	0.999	0.999	0.001	0.030	0.024	0.001	0.005	0.004
**51**	91	1.000	1.000	1.000	0.001	0.002	0.004	0.001	0.002	0.004
**52**	444	0.994	0.892	0.862	0.061	0.253	0.287	0.060	0.148	0.167
**53**	91	0.903	0.949	0.971	0.305	0.222	0.168	0.112	0.071	0.096
**54**	208	1.000	0.998	1.000	0.001	0.067	0.001	0.001	0.009	0.001
**55**	105	1.000	1.000	1.000	0.001	0.013	0.001	0.001	0.002	0.001
**56**	96	1.000	0.963	0.989	0.002	0.237	0.128	0.001	0.232	0.123

**Reaction dataset ID**	**Count (reactant pairs)**	**R2**			**RMSE**			**MAE**		
		**(1)**	**(2)**	**(3)**	**(1)**	**(2)**	**(3)**	**(1)**	**(2)**	**(3)**
**57**	120	0.986	0.983	0.968	0.164	0.182	0.251	0.048	0.081	0.144
**58**	364	0.912	0.919	0.948	0.332	0.320	0.255	0.030	0.131	0.074
**59**	724	0.996	0.973	0.993	0.064	0.161	0.081	0.014	0.127	0.041
**60**	669	0.965	0.861	0.711	0.217	0.433	0.626	0.203	0.266	0.295

Table S6 Prediction accuracies with the best hyperparameters for the test datasets (Product-based without augmentation). (1) SVR-PK, **(2)** SVR-SK, **(3)** SVR-concatECFP, **(4)** SVR-baseline, **(5)** MolCLR.

**Reaction dataset ID**	**Count (reactant pairs)**	**Count (products)**	**R2**					**RMSE**					**MAE**				
			**(1)**	**(2)**	**(3)**	**(4)**	**(5)**	**(1)**	**(2)**	**(3)**	**(4)**	**(5)**	**(1)**	**(2)**	**(3)**	**(4)**	**(5)**
**1**	38	36	0.567	0.548	0.568	0.626	0.612	1.116	1.140	1.115	1.038	1.057	0.717	0.776	0.729	0.655	0.730
**2**	104	63	0.700	0.712	0.758	0.745	0.503	0.694	0.679	0.622	0.639	0.893	0.521	0.472	0.448	0.456	0.715
**3**	135	76	0.338	0.345	0.412	0.348	-0.009	0.731	0.727	0.689	0.725	0.903	0.577	0.561	0.534	0.568	0.735
**4**	117	99	0.624	0.713	0.726	0.683	0.484	0.859	0.750	0.733	0.788	1.005	0.616	0.549	0.534	0.563	0.756
**5**	494	424	0.590	0.618	0.626	0.614	0.521	0.738	0.713	0.705	0.717	0.798	0.547	0.527	0.514	0.523	0.587
**6**	94	84	0.313	0.397	0.378	0.392	0.507	0.748	0.701	0.711	0.704	0.634	0.547	0.517	0.540	0.509	0.498
**7**	838	778	0.580	0.557	0.595	0.603	0.474	0.666	0.683	0.654	0.647	0.745	0.490	0.503	0.477	0.481	0.543
**8**	47	46	0.318	0.285	0.278	0.276	0.323	0.856	0.876	0.881	0.882	0.853	0.671	0.665	0.673	0.666	0.660
**9**	49	41	0.333	0.170	0.280	0.350	-0.860	0.658	0.734	0.683	0.650	1.099	0.498	0.560	0.524	0.478	0.928
**10**	56	51	0.064	-0.049	0.006	0.114	-0.080	0.864	0.915	0.890	0.840	0.928	0.703	0.732	0.708	0.674	0.751
**11**	55	51	0.152	0.235	0.155	0.177	0.063	0.785	0.746	0.784	0.774	0.825	0.638	0.566	0.613	0.604	0.684
**12**	69	67	0.386	0.505	0.510	0.472	0.436	0.843	0.757	0.753	0.781	0.808	0.660	0.597	0.612	0.625	0.633
**13**	115	114	0.418	0.404	0.450	0.441	0.093	0.810	0.820	0.787	0.794	1.011	0.649	0.655	0.629	0.635	0.813
**14**	269	257	0.446	0.454	0.509	0.514	0.423	0.833	0.828	0.785	0.781	0.851	0.664	0.652	0.619	0.616	0.659
**15**	336	171	0.568	0.548	0.562	0.587	0.516	0.787	0.805	0.792	0.770	0.833	0.617	0.637	0.615	0.598	0.651
**16**	38	38	0.492	0.492	0.489	0.490	0.416	0.738	0.738	0.740	0.739	0.791	0.579	0.579	0.583	0.579	0.647
**17**	40	38	0.097	0.048	0.074	0.211	0.162	0.919	0.943	0.931	0.859	0.885	0.670	0.644	0.643	0.621	0.676
**18**	45	42	0.746	0.746	0.748	0.752	0.674	0.900	0.900	0.896	0.888	1.019	0.753	0.753	0.749	0.745	0.784
**19**	47	41	0.518	0.481	0.544	0.537	0.363	0.707	0.734	0.688	0.693	0.813	0.552	0.572	0.529	0.546	0.675
**20**	50	39	0.298	0.146	0.212	0.210	-0.067	0.576	0.635	0.610	0.611	0.710	0.430	0.490	0.465	0.468	0.536
**21**	60	52	0.232	0.294	0.366	0.374	-0.253	0.831	0.797	0.755	0.750	1.062	0.662	0.647	0.598	0.598	0.859
**22**	127	84	0.419	0.510	0.538	0.512	0.401	0.898	0.825	0.801	0.823	0.912	0.701	0.629	0.597	0.617	0.657
**23**	54	45	0.619	0.620	0.616	0.581	0.482	0.664	0.663	0.666	0.696	0.774	0.511	0.509	0.513	0.510	0.569

Reaction dataset ID	Count (reactant pairs)	Count (products)	R2					RMSE					MAE				
			(1)	(2)	(3)	(4)	(5)	(1)	(2)	(3)	(4)	(5)	(1)	(2)	(3)	(4)	(5)
**24**	52	34	0.223	0.347	0.386	0.417	0.139	0.899	0.824	0.799	0.778	0.946	0.675	0.642	0.616	0.609	0.712
**25**	114	68	0.481	0.414	0.491	0.454	0.212	0.873	0.928	0.865	0.896	1.076	0.691	0.706	0.656	0.676	0.855
**26**	375	350	0.693	0.720	0.710	0.695	0.585	0.586	0.560	0.569	0.584	0.681	0.421	0.412	0.405	0.414	0.499
**27**	37	33	0.217	0.217	0.218	0.205	0.306	0.967	0.967	0.966	0.974	0.910	0.715	0.714	0.715	0.714	0.618
**28**	30	25	-0.021	0.254	0.234	0.341	-0.168	1.032	0.882	0.894	0.829	1.104	0.889	0.768	0.766	0.715	0.887
**29**	54	33	-0.028	0.051	-0.006	-0.023	-0.444	0.677	0.651	0.670	0.676	0.803	0.558	0.540	0.539	0.537	0.613
**30**	75	63	0.398	0.359	0.395	0.393	0.153	0.748	0.772	0.749	0.751	0.887	0.571	0.606	0.583	0.573	0.704
**31**	118	113	0.574	0.545	0.560	0.563	0.513	0.660	0.682	0.671	0.668	0.706	0.478	0.483	0.473	0.494	0.538
**32**	37	37	0.351	0.351	0.350	0.370	0.206	0.759	0.759	0.760	0.748	0.840	0.630	0.630	0.631	0.617	0.681
**33**	52	51	0.720	0.732	0.721	0.721	0.244	0.820	0.801	0.818	0.818	1.346	0.662	0.643	0.660	0.664	0.992
**34**	38	31	0.314	0.479	0.365	0.375	0.132	1.003	0.874	0.964	0.957	1.128	0.772	0.690	0.747	0.713	0.778
**35**	54	38	0.568	0.597	0.576	0.595	0.470	0.776	0.749	0.769	0.751	0.859	0.578	0.574	0.590	0.560	0.642
**36**	113	99	0.461	0.462	0.484	0.499	0.376	0.956	0.955	0.936	0.922	1.029	0.780	0.793	0.769	0.759	0.863
**37**	165	113	0.427	0.478	0.471	0.447	0.242	0.882	0.843	0.848	0.867	1.015	0.618	0.594	0.603	0.618	0.658
**38**	124	121	0.456	0.486	0.501	0.544	0.257	0.852	0.828	0.816	0.780	0.996	0.682	0.641	0.624	0.591	0.740
**39**	32	31	0.426	0.418	0.495	0.435	0.293	0.616	0.620	0.577	0.611	0.684	0.483	0.494	0.452	0.496	0.485
**40**	39	39	0.690	0.777	0.683	0.668	0.733	1.541	1.307	1.557	1.595	1.429	1.067	0.984	1.072	1.091	1.090
**41**	33	30	0.327	0.416	0.409	0.447	-0.072	0.811	0.756	0.760	0.736	1.024	0.630	0.562	0.561	0.550	0.748
**42**	56	47	0.302	0.231	0.277	0.310	0.240	0.874	0.917	0.890	0.869	0.912	0.710	0.706	0.678	0.652	0.702
**43**	89	67	0.489	0.524	0.547	0.542	0.508	0.683	0.660	0.643	0.647	0.670	0.447	0.422	0.395	0.411	0.444
**44**	30	30	0.231	0.224	0.237	0.297	-0.255	0.602	0.604	0.599	0.575	0.768	0.462	0.465	0.455	0.430	0.594
**45**	30	30	0.189	0.247	0.282	0.270	0.263	0.464	0.447	0.437	0.440	0.442	0.357	0.346	0.339	0.345	0.372
**46**	40	37	0.091	0.065	0.083	0.129	-0.646	0.955	0.968	0.958	0.934	1.284	0.704	0.692	0.696	0.698	0.903
**47**	48	31	0.386	0.272	0.325	0.338	0.087	0.599	0.652	0.628	0.622	0.731	0.440	0.503	0.480	0.458	0.565
**48**	59	42	0.389	0.424	0.423	0.354	-0.034	0.718	0.698	0.698	0.739	0.934	0.564	0.558	0.548	0.587	0.694
**49**	43	39	0.327	0.376	0.384	0.371	0.060	0.708	0.682	0.677	0.684	0.837	0.559	0.526	0.527	0.554	0.722

Reaction dataset ID	Count (reactant pairs)	Count (products)	R2					RMSE					MAE				
			(1)	(2)	(3)	(4)	(5)	(1)	(2)	(3)	(4)	(5)	(1)	(2)	(3)	(4)	(5)
**50**	65	63	0.550	0.673	0.620	0.625	0.677	0.713	0.607	0.655	0.651	0.604	0.591	0.503	0.553	0.547	0.500
**51**	86	82	0.170	0.232	0.182	0.169	-0.129	0.719	0.692	0.714	0.719	0.839	0.570	0.565	0.577	0.570	0.626
**52**	236	112	0.643	0.652	0.642	0.628	0.565	0.597	0.589	0.598	0.609	0.659	0.461	0.432	0.440	0.452	0.542
**53**	121	96	0.452	0.408	0.442	0.442	0.309	0.931	0.967	0.939	0.940	1.045	0.708	0.736	0.718	0.741	0.807
**54**	126	101	0.677	0.677	0.678	0.667	0.523	0.780	0.780	0.780	0.793	0.949	0.622	0.622	0.621	0.634	0.739
**55**	124	105	0.316	0.338	0.354	0.340	0.284	0.849	0.836	0.825	0.834	0.869	0.593	0.605	0.577	0.577	0.677
**56**	119	111	0.520	0.544	0.513	0.537	0.393	1.024	0.998	1.031	1.005	1.151	0.666	0.651	0.648	0.645	0.700
**57**	124	112	0.427	0.436	0.435	0.391	0.365	1.142	1.133	1.133	1.177	1.202	0.727	0.759	0.723	0.710	0.779
**58**	238	170	0.315	0.316	0.312	0.368	0.320	0.971	0.970	0.973	0.933	0.968	0.642	0.661	0.629	0.614	0.737
**59**	386	193	0.471	0.498	0.507	0.503	0.301	0.835	0.813	0.805	0.809	0.960	0.607	0.599	0.594	0.583	0.667
**60**	612	458	0.621	0.625	0.632	0.615	0.510	0.802	0.798	0.790	0.809	0.912	0.616	0.616	0.592	0.621	0.657

Table S7 Prediction accuracies with the best hyperparameters for the test datasets (Product-based with augmentation). (1) SVR-PK, **(2)** SVR-SK, **(3)** SVR-concatECFP. SVR-baseline and MolCLR results are the same as in **Table S6** and therefore omitted. Only the prediction accuracies of the reaction datasets for which data augmentation could be performed are reported.

**Reaction dataset ID**	**Count (reactant pairs)**	**R2**			**RMSE**			**MAE**		
		**(1)**	**(2)**	**(3)**	**(1)**	**(2)**	**(3)**	**(1)**	**(2)**	**(3)**
**2**	104	0.703	0.731	0.764	0.690	0.656	0.615	0.516	0.460	0.441
**3**	135	0.426	0.325	0.409	0.681	0.738	0.691	0.545	0.577	0.537
**4**	117	0.630	0.714	0.720	0.851	0.748	0.740	0.617	0.554	0.534
**5**	494	0.591	0.617	0.612	0.737	0.714	0.719	0.545	0.528	0.524
**6**	94	0.330	0.377	0.374	0.738	0.712	0.714	0.545	0.522	0.529
**7**	838	0.583	0.564	0.600	0.663	0.678	0.649	0.489	0.501	0.474
**9**	49	0.336	0.172	0.271	0.656	0.733	0.688	0.509	0.564	0.530
**10**	56	0.070	-0.069	0.005	0.861	0.923	0.891	0.693	0.731	0.701
**11**	55	0.154	0.135	0.147	0.784	0.793	0.787	0.636	0.636	0.618
**12**	69	0.359	0.401	0.505	0.861	0.833	0.757	0.673	0.644	0.614
**13**	115	0.400	0.401	0.435	0.823	0.822	0.798	0.655	0.657	0.631
**14**	269	0.452	0.467	0.531	0.829	0.818	0.767	0.659	0.644	0.602
**15**	336	0.585	0.549	0.569	0.771	0.804	0.786	0.607	0.634	0.611
**22**	127	0.439	0.551	0.553	0.883	0.790	0.788	0.682	0.592	0.586
**23**	54	0.619	0.620	0.616	0.664	0.663	0.666	0.511	0.510	0.514
**24**	52	0.226	0.376	0.395	0.897	0.806	0.793	0.670	0.615	0.607
**25**	114	0.475	0.334	0.473	0.879	0.990	0.880	0.679	0.747	0.661
**26**	375	0.688	0.708	0.707	0.590	0.571	0.572	0.427	0.418	0.414
**27**	37	0.214	0.215	0.215	0.968	0.968	0.968	0.716	0.715	0.716
**28**	30	0.098	0.274	0.302	0.970	0.870	0.853	0.850	0.760	0.729
**29**	54	0.001	0.056	-0.006	0.668	0.649	0.670	0.549	0.526	0.534
**31**	118	0.584	0.550	0.571	0.652	0.678	0.662	0.474	0.487	0.468

Reaction dataset ID	Count (reactant pairs)	R2			RMSE			MAE		
		(1)	(2)	(3)	(1)	(2)	(3)	(1)	(2)	(3)
**36**	113	0.462	0.493	0.488	0.956	0.928	0.932	0.777	0.759	0.757
**37**	165	0.420	0.455	0.461	0.888	0.861	0.856	0.624	0.616	0.617
**38**	124	0.477	0.540	0.516	0.835	0.783	0.804	0.669	0.592	0.621
**42**	56	0.282	0.213	0.257	0.886	0.928	0.902	0.711	0.712	0.675
**43**	89	0.524	0.512	0.556	0.659	0.667	0.637	0.426	0.422	0.388
**46**	40	0.079	-0.003	0.080	0.961	1.003	0.960	0.707	0.725	0.698
**47**	48	0.386	0.284	0.311	0.599	0.647	0.635	0.437	0.493	0.482
**48**	59	0.389	0.432	0.428	0.718	0.693	0.695	0.564	0.548	0.544
**49**	43	0.327	0.373	0.381	0.708	0.683	0.679	0.559	0.526	0.529
**52**	236	0.649	0.651	0.654	0.592	0.591	0.588	0.448	0.429	0.437
**53**	121	0.454	0.411	0.449	0.929	0.965	0.933	0.706	0.732	0.712
**54**	126	0.666	0.666	0.666	0.794	0.794	0.794	0.639	0.637	0.639
**55**	124	0.321	0.346	0.368	0.846	0.831	0.816	0.593	0.605	0.575
**56**	119	0.520	0.496	0.513	1.024	1.049	1.032	0.666	0.659	0.648
**57**	124	0.471	0.517	0.444	1.097	1.048	1.125	0.717	0.741	0.691
**58**	238	0.313	0.314	0.315	0.972	0.972	0.971	0.643	0.666	0.633
**59**	386	0.481	0.516	0.528	0.827	0.798	0.789	0.594	0.579	0.577
**60**	612	0.616	0.603	0.629	0.808	0.821	0.794	0.625	0.645	0.595

Table S8 Prediction accuracies with the best hyperparameters for the test datasets (Reactant-based without augmentation). (1) SVR-PK, **(2)** SVR-SK, **(3)** SVR-concatECFP, **(4)** SVR-baseline, **(5)** MolCLR.

Reaction dataset ID	Count (reactant pairs)	Count (products)	R2					RMSE					MAE				
			(1)	(2)	(3)	(4)	(5)	(1)	(2)	(3)	(4)	(5)	(1)	(2)	(3)	(4)	(5)
**1**	41	41	-0.341	-0.423	-0.502	-0.520	-0.478	1.981	2.041	2.097	2.109	2.079	1.374	1.424	1.462	1.430	1.428
**2**	52	50	-0.176	0.004	-0.050	0.515	0.210	1.426	1.312	1.348	0.916	1.169	0.933	0.900	0.901	0.718	0.908
**3**	70	68	-0.334	-0.164	-0.202	-0.040	-0.728	0.916	0.855	0.869	0.808	1.042	0.680	0.634	0.647	0.612	0.851
**4**	86	78	-0.255	-0.325	-0.351	-0.794	-2.987	1.595	1.639	1.655	1.907	2.844	1.318	1.349	1.348	1.530	2.309
**5**	336	329	0.153	0.223	0.274	0.334	0.018	0.993	0.950	0.919	0.880	1.069	0.811	0.772	0.749	0.706	0.869
**6**	71	66	-0.605	-0.722	-0.730	-0.291	-0.418	1.124	1.164	1.167	1.008	1.056	0.907	0.950	0.938	0.773	0.856
**7**	509	501	0.010	0.146	0.073	0.075	-0.363	0.958	0.890	0.927	0.926	1.124	0.745	0.693	0.728	0.725	0.880
**8**	25	25	-0.153	-0.104	-0.156	-0.413	-3.472	1.029	1.006	1.030	1.139	2.026	0.924	0.897	0.935	1.024	1.804
**9**	26	23	0.061	-0.272	-0.083	0.313	0.332	0.642	0.748	0.690	0.550	0.542	0.505	0.586	0.529	0.424	0.419
**10**	41	40	-0.281	-0.388	-0.309	-0.341	-0.269	0.940	0.979	0.951	0.962	0.936	0.796	0.812	0.790	0.799	0.700
**11**	50	49	-0.145	-0.251	-0.171	-0.117	-1.314	1.147	1.199	1.159	1.133	1.630	0.873	0.916	0.903	0.859	1.197
**12**	42	42	-0.530	-0.080	-0.463	-0.559	-1.175	1.490	1.252	1.457	1.504	1.777	1.250	1.075	1.228	1.259	1.501
**13**	39	39	-0.113	-0.381	-0.399	-0.458	-1.158	1.324	1.475	1.484	1.515	1.844	1.060	1.123	1.134	1.162	1.374
**14**	164	159	0.076	0.128	0.185	0.110	0.003	1.130	1.097	1.061	1.109	1.173	0.879	0.862	0.813	0.871	0.876
**15**	152	140	-0.526	-0.036	-0.034	0.416	0.358	1.200	0.989	0.988	0.742	0.779	1.016	0.811	0.806	0.562	0.583
**17**	31	31	-1.031	-0.827	-0.807	-2.401	-0.774	0.729	0.691	0.688	0.943	0.681	0.609	0.559	0.552	0.833	0.584
**19**	23	20	-0.044	-0.011	0.038	0.213	0.227	0.809	0.796	0.777	0.703	0.696	0.712	0.671	0.657	0.647	0.603
**20**	29	29	0.010	0.094	0.080	-0.048	-0.585	0.791	0.757	0.763	0.814	1.001	0.666	0.642	0.646	0.652	0.828
**21**	39	39	-0.476	-0.563	-0.327	-0.452	-0.960	1.074	1.105	1.019	1.066	1.238	0.810	0.829	0.769	0.800	1.007
**22**	63	56	-0.020	-0.030	-0.021	-0.015	-0.344	1.375	1.382	1.375	1.371	1.578	0.997	1.007	1.002	1.024	1.215
**24**	38	25	0.075	0.069	-0.011	-0.089	-0.889	0.560	0.562	0.586	0.608	0.801	0.441	0.432	0.442	0.449	0.580
**25**	44	42	-0.314	-0.423	-0.465	0.357	0.409	1.260	1.311	1.330	0.881	0.845	1.037	1.099	1.098	0.721	0.697
**26**	243	240	0.063	0.307	0.271	0.411	0.263	1.109	0.954	0.978	0.879	0.984	0.900	0.742	0.773	0.695	0.779

**Reaction dataset ID**	**Count (reactant pairs)**	**Count (products)**	**R2**					**RMSE**					**MAE**				
			**(1)**	**(2)**	**(3)**	**(4)**	**(5)**	**(1)**	**(2)**	**(3)**	**(4)**	**(5)**	**(1)**	**(2)**	**(3)**	**(4)**	**(5)**
**28**	28	17	-0.019	-0.010	0.089	0.264	-0.642	0.978	0.974	0.925	0.831	1.241	0.849	0.836	0.832	0.776	1.115
**29**	25	24	-0.099	-0.111	-0.133	-0.180	-0.170	0.768	0.772	0.780	0.795	0.792	0.626	0.626	0.638	0.693	0.681
**30**	58	56	0.002	-0.053	-0.024	-0.105	-0.336	0.980	1.007	0.993	1.031	1.134	0.843	0.863	0.854	0.888	0.961
**31**	89	87	0.214	0.286	0.403	0.298	0.304	1.013	0.965	0.883	0.957	0.953	0.827	0.796	0.740	0.779	0.747
**34**	28	24	0.018	0.081	0.034	0.115	0.347	1.115	1.079	1.106	1.059	0.909	0.902	0.839	0.891	0.799	0.708
**35**	40	38	-0.614	-0.811	-0.942	-0.883	-0.145	1.609	1.705	1.766	1.739	1.356	1.328	1.403	1.451	1.436	1.186
**36**	82	80	0.108	0.174	0.177	0.149	-0.125	1.058	1.018	1.016	1.034	1.188	0.874	0.818	0.828	0.833	0.996
**37**	88	73	-0.007	0.051	0.112	0.025	0.077	0.958	0.930	0.899	0.942	0.917	0.615	0.585	0.572	0.592	0.658
**38**	114	114	0.070	0.198	0.183	0.043	-0.540	0.807	0.750	0.757	0.819	1.039	0.650	0.594	0.587	0.623	0.848
**39**	20	20	0.129	0.047	0.046	0.079	-0.279	0.599	0.627	0.627	0.616	0.726	0.463	0.489	0.492	0.485	0.562
**41**	36	35	0.138	0.254	0.274	0.311	0.225	1.025	0.954	0.940	0.916	0.972	0.800	0.705	0.715	0.729	0.758
**42**	39	39	-0.268	-0.339	-0.301	-0.474	-2.080	1.161	1.193	1.176	1.252	1.809	0.927	1.001	0.978	1.039	1.577
**43**	44	39	-0.014	0.001	-0.004	0.009	-0.116	1.454	1.444	1.447	1.438	1.526	1.034	1.030	1.034	1.037	1.115
**44**	26	26	0.121	0.064	0.056	-0.016	-0.012	0.572	0.591	0.593	0.615	0.614	0.453	0.482	0.486	0.505	0.486
**45**	15	15	0.108	0.283	0.265	0.255	0.358	0.647	0.580	0.587	0.591	0.549	0.491	0.434	0.429	0.460	0.409
**46**	35	31	-0.022	-0.026	-0.012	0.161	0.235	1.171	1.174	1.166	1.061	1.013	0.924	0.938	0.934	0.751	0.823
**47**	39	38	0.082	0.033	0.090	0.134	-0.190	0.700	0.718	0.697	0.680	0.796	0.540	0.628	0.600	0.553	0.678
**48**	40	32	-0.146	-0.182	-0.131	-0.296	-0.810	0.840	0.854	0.835	0.894	1.056	0.653	0.669	0.662	0.697	0.857
**49**	33	33	-0.145	-0.044	-0.126	0.073	0.236	0.910	0.869	0.902	0.819	0.743	0.775	0.752	0.774	0.705	0.642
**50**	38	37	0.005	0.016	-0.033	0.034	-1.074	0.559	0.556	0.570	0.551	0.807	0.474	0.473	0.477	0.457	0.670
**51**	73	71	0.035	-0.084	-0.055	0.135	-0.057	0.741	0.785	0.775	0.702	0.775	0.602	0.642	0.631	0.567	0.641
**52**	81	73	0.166	0.293	0.296	0.387	-0.159	0.695	0.640	0.639	0.596	0.820	0.569	0.523	0.516	0.470	0.640
**53**	57	46	-0.134	-0.168	-0.128	-0.036	-0.076	1.434	1.456	1.430	1.371	1.397	1.114	1.121	1.106	1.064	1.166
**55**	63	62	0.258	0.192	0.391	0.230	-0.160	0.805	0.840	0.729	0.820	1.007	0.662	0.693	0.622	0.693	0.860
**56**	72	72	-0.002	-0.053	-0.042	0.013	-0.207	1.844	1.890	1.880	1.830	2.023	1.161	1.253	1.225	1.240	1.537
**57**	92	90	0.054	0.196	0.143	0.202	0.194	0.993	0.915	0.945	0.912	0.916	0.847	0.775	0.811	0.752	0.761

Reaction dataset ID	Count (reactant pairs)	Count (products)	R2					RMSE					MAE				
			(1)	(2)	(3)	(4)	(5)	(1)	(2)	(3)	(4)	(5)	(1)	(2)	(3)	(4)	(5)
**58**	109	95	0.009	-0.130	-0.072	-0.283	-0.185	1.183	1.263	1.230	1.345	1.293	0.901	0.990	0.931	1.029	0.970
**59**	118	116	0.043	-0.018	0.056	0.274	-0.203	0.993	1.024	0.986	0.865	1.113	0.829	0.848	0.826	0.701	0.885
**60**	221	214	-0.109	-0.668	-0.411	-0.344	-0.325	0.996	1.221	1.123	1.096	1.088	0.724	0.949	0.854	0.799	0.809

Table S9 Prediction accuracies with the best hyperparameters for the test datasets (Reactant-based with augmentation). (1) SVR-PK, **(2)** SVR-SK, **(3)** SVR-concatECFP**.** SVR-baseline and MolCLR results are the same as in **Table S8** and therefore omitted. Only the prediction accuracies of the reaction datasets for which data augmentation could be performed are reported.

**Reaction dataset**	**Count**		**R2**			**RMSE**			**MAE**	
**ID**	**(reactant pairs)**	**(1)**	**(2)**	**(3)**	**(1)**	**(2)**	**(3)**	**(1)**	**(2)**	**(3)**
**1**	41	-0.329	-0.418	-0.498	1.972	2.037	2.094	1.367	1.414	1.452
**2**	52	0.512	0.502	0.545	0.918	0.928	0.887	0.703	0.754	0.696
**3**	70	-0.167	-0.148	-0.084	0.856	0.849	0.825	0.645	0.635	0.617
**4**	86	-1.026	-1.233	-0.928	2.027	2.128	1.977	1.683	1.783	1.641
**5**	336	0.261	0.285	0.315	0.927	0.912	0.893	0.735	0.690	0.693
**6**	71	-0.796	-0.892	-0.831	1.189	1.220	1.200	0.923	0.956	0.934
**7**	509	0.057	0.123	0.063	0.935	0.902	0.932	0.718	0.701	0.726
**9**	26	0.157	0.161	0.139	0.609	0.607	0.615	0.456	0.457	0.470
**10**	41	-0.229	-0.317	-0.282	0.921	0.954	0.941	0.774	0.789	0.783
**11**	50	-0.105	-0.124	-0.123	1.127	1.136	1.136	0.877	0.873	0.874
**12**	42	-0.508	-0.091	-0.466	1.480	1.258	1.459	1.242	1.074	1.226
**13**	39	-0.113	-0.372	-0.392	1.324	1.470	1.481	1.060	1.114	1.129
**14**	164	0.078	0.136	0.189	1.129	1.092	1.058	0.876	0.854	0.808
**15**	152	0.411	0.419	0.448	0.745	0.741	0.722	0.587	0.581	0.557
**17**	31	-2.091	-2.258	-2.305	0.899	0.923	0.930	0.789	0.814	0.819
**19**	23	-0.199	0.191	-0.005	0.867	0.712	0.794	0.771	0.618	0.705
**20**	29	0.097	0.011	-0.004	0.755	0.791	0.797	0.639	0.676	0.671
**21**	39	-0.481	-0.371	-0.546	1.076	1.035	1.099	0.807	0.794	0.823
**22**	63	-0.057	-0.108	-0.069	1.400	1.433	1.407	1.029	1.053	1.028
**24**	38	-0.320	-0.526	-0.202	0.669	0.720	0.639	0.508	0.522	0.472
**25**	44	0.420	0.140	0.296	0.837	1.019	0.922	0.668	0.814	0.738
**26**	243	0.070	0.302	0.268	1.105	0.957	0.980	0.894	0.752	0.775
**28**	28	0.367	0.239	0.229	0.771	0.845	0.851	0.716	0.756	0.764

Reaction dataset ID	Count (reactant pairs)	R2 (1)	R2 (2)	R2 (3)	RMSE (1)	RMSE (2)	RMSE (3)	MAE (1)	MAE (2)	MAE (3)
**29**	25	-0.006	-0.212	-0.155	0.734	0.806	0.787	0.634	0.709	0.682
**30**	58	-0.037	-0.048	-0.032	0.999	1.004	0.997	0.865	0.860	0.868
**31**	89	0.253	0.280	0.385	0.987	0.969	0.895	0.804	0.801	0.741
**34**	28	0.018	0.058	0.048	1.115	1.092	1.098	0.892	0.823	0.862
**35**	40	-0.302	-0.526	-0.768	1.446	1.565	1.685	1.223	1.281	1.380
**36**	82	0.173	0.193	0.216	1.018	1.006	0.992	0.835	0.807	0.807
**37**	88	-0.032	-0.111	0.049	0.969	1.006	0.931	0.628	0.664	0.628
**38**	114	0.164	0.280	0.251	0.766	0.711	0.725	0.590	0.552	0.555
**39**	20	0.119	0.094	0.086	0.603	0.611	0.614	0.467	0.473	0.477
**41**	36	0.181	0.317	0.332	0.999	0.912	0.902	0.789	0.681	0.696
**42**	39	-0.182	-0.214	-0.203	1.121	1.136	1.131	0.902	0.944	0.937
**43**	44	-0.019	-0.029	-0.020	1.458	1.465	1.459	1.045	1.056	1.050
**46**	35	0.187	0.197	0.196	1.045	1.038	1.039	0.736	0.732	0.744
**47**	39	0.065	0.070	0.089	0.706	0.704	0.697	0.528	0.608	0.595
**48**	40	-0.154	-0.162	-0.184	0.843	0.846	0.854	0.658	0.668	0.674
**49**	33	-0.117	0.043	-0.076	0.899	0.832	0.882	0.770	0.727	0.751
**50**	38	0.017	0.027	-0.016	0.556	0.553	0.565	0.475	0.473	0.474
**51**	73	0.027	-0.097	-0.070	0.744	0.790	0.780	0.604	0.646	0.635
**52**	81	0.404	0.475	0.472	0.588	0.552	0.553	0.443	0.430	0.420
**53**	57	-0.020	-0.054	-0.136	1.360	1.383	1.436	1.084	1.115	1.112
**55**	63	0.244	0.213	0.281	0.813	0.829	0.792	0.687	0.710	0.671
**56**	72	0.017	0.040	0.004	1.826	1.804	1.838	1.152	1.099	1.162
**57**	92	0.047	0.141	0.117	0.997	0.946	0.959	0.856	0.812	0.829
**58**	109	-0.008	-0.009	-0.079	1.192	1.193	1.234	0.917	0.947	0.936
**59**	118	0.292	0.223	0.226	0.854	0.895	0.893	0.696	0.734	0.732
**60**	221	0.048	-0.198	-0.202	0.923	1.035	1.037	0.678	0.785	0.738
